# Unbiased analysis of obesity related, fat depot specific changes of adipocyte volumes and numbers using light sheet fluorescence microscopy

**DOI:** 10.1371/journal.pone.0248594

**Published:** 2021-03-16

**Authors:** Natalie Theobalt, Isabel Hofmann, Sonja Fiedler, Simone Renner, Georg Dhom, Annette Feuchtinger, Axel Walch, Martin Hrabĕ de Angelis, Eckhard Wolf, Rüdiger Wanke, Andreas Blutke

**Affiliations:** 1 Institute of Veterinary Pathology at the Center for Clinical Veterinary Medicine, Ludwig-Maximilians-Universität München, Munich, Germany; 2 Gene Center and Department of Veterinary Sciences, Chair for Molecular Animal Breeding and Biotechnology, Ludwig-Maximilians-Universität München, Munich, Germany; 3 Department of Veterinary Sciences, Center for Innovative Medical Models (CiMM), Ludwig-Maximilians-Universität München, Oberschleißheim, Germany; 4 German Center for Diabetes Research (DZD), Neuherberg, Germany; 5 Research Unit Analytical Pathology, Helmholtz Zentrum München, Neuherberg, Germany; 6 Institute of Experimental Genetics, Helmholtz Zentrum München, Neuherberg, Germany; 7 Laboratory for Functional Genome Analysis (LAFUGA), Gene Center, Ludwig-Maximilians-Universität München, Munich, Germany; University of Campinas, BRAZIL

## Abstract

In translational obesity research, objective assessment of adipocyte sizes and numbers is essential to characterize histomorphological alterations linked to obesity, and to evaluate the efficacies of experimental medicinal or dietetic interventions. Design-based quantitative stereological techniques based on the analysis of 2D-histological sections provide unbiased estimates of relevant 3D-parameters of adipocyte morphology, but often involve complex and time-consuming tissue processing and analysis steps. Here we report the application of direct 3D light sheet fluorescence microscopy (LSFM) for effective and accurate analysis of adipocyte volumes and numbers in optically cleared adipose tissue samples from a porcine model of diet-induced obesity (DIO). Subcutaneous and visceral adipose tissue samples from DIO-minipigs and lean controls were systematically randomly sampled, optically cleared with 3DISCO (3-dimensional imaging of solvent cleared organs), stained with eosin, and subjected to LSFM for detection of adipocyte cell membrane autofluorescence. Individual adipocytes were unbiasedly sampled in digital 3D reconstructions of the adipose tissue samples, and their individual cell volumes were directly measured by automated digital image analysis. Adipocyte numbers and mean volumes obtained by LSFM analysis did not significantly differ from the corresponding values obtained by unbiased quantitative stereological analysis techniques performed on the same samples, thus proving the applicability of LSFM for efficient analysis of relevant morphological adipocyte parameters. The results of the present study demonstrate an adipose tissue depot specific plasticity of adipocyte growth responses to nutrient oversupply. This was characterized by an exclusively hypertrophic growth of visceral adipocytes, whereas adipocytes in subcutaneous fat tissue depots also displayed a marked (hyperplastic) increase in cell number. LSFM allows for accurate and efficient determination of relevant quantitative morphological adipocyte parameters. The applied stereological methods and LSFM protocols are described in detail and can serve as a guideline for unbiased quantitative morphological analyses of adipocytes in other studies and species.

## Introduction

Worldwide, the prevalence of obesity and its sequelae, such as the metabolic syndrome (MetS), is constantly rising [1]. Particularly visceral adiposity and the development of an associated local and systemic inflammatory response are key determinants in the pathogenesis of the human MetS [2,3]. In translational research, various experimental obesity models of different species are used to study obesity related metabolic derangements and their role in the pathogenesis of adiposity related diseases [4]. In addition to “classic” rodent models of adiposity, also diverse large animal models (LAM) are increasingly used due to their physiological and metabolic similarities to humans [4,5]. Göttingen minipigs (GM) with diet induced obesity (DIO) exhibit severe subcutaneous and visceral adiposity, diverse metabolic dysregulations, such as disturbed glucose tolerance, and develop manifest histopathological adipose tissue inflammation, which is restricted to the visceral adipose tissue [6]. Understanding of the pathogenesis-relevant processes in the adipose tissue during development of obesity and the MetS requires a joint consideration of histomorphological changes, such as adipocyte hypertrophy/hyperplasia and alterations of the cellular tissue composition due to infiltration of inflammatory cells, as well as associated molecular alterations, such as differential abundances of inflammatory cytokines and lipokines in the adipose tissue. Relevant morphological changes of adipocytes in developing or manifest obesity are best characterized by unbiased quantitative analyses of the number, the volume, and the mean volume of adipocytes in a defined adipose tissue depot. These parameters allow for an objective identification, definition, and comparison of already subtle quantitative morphological alterations of cells [7–9], such as adipocytes, *e*.*g*., by differentiation of hyperplastic and hypertrophic adipocyte growth patterns [10,11]. Quantitative parameters of adipocyte morphology can thus also help to interpret the results of *e*.*g*., lipidomic, transcriptomic, proteomic, or metabolomic profiling analyses of adipose tissue samples [12]. A thorough analysis of obesity related changes in adipose tissue samples should therefore also include quantitative morphological analyses. The generally accepted “gold-standard” for quantitative morphological analyses in histological tissue sections are so-called “unbiased quantitative stereological” analysis methods, combined with (systematic) random sampling methods [13,14]. Unbiased quantitative stereological analyses are capable to provide assumption-free, accurate and precise estimates of quantitative morphological parameters (*i*.*e*., volumes, surfaces, lengths, numbers) of the examined tissue structures of interest with known error probabilities by analysis of adequately sampled and oriented histological sections, using appropriate stereological probes [7–9,15,16]. In various biomedical research disciplines, such as neurosciences or nephrology, unbiased quantitative stereological analysis methods have become an indispensable component in studies involving quantitative analyses of histomorphological tissue features [13,17,18]. However, application of unbiased quantitative stereological methods is often regarded quite time consuming and cumbersome, since these methods usually require the observance of strict sampling designs and often use special histotechniques, such as plastic embedding and preparation of thin sections [16].

**Fig 1:**
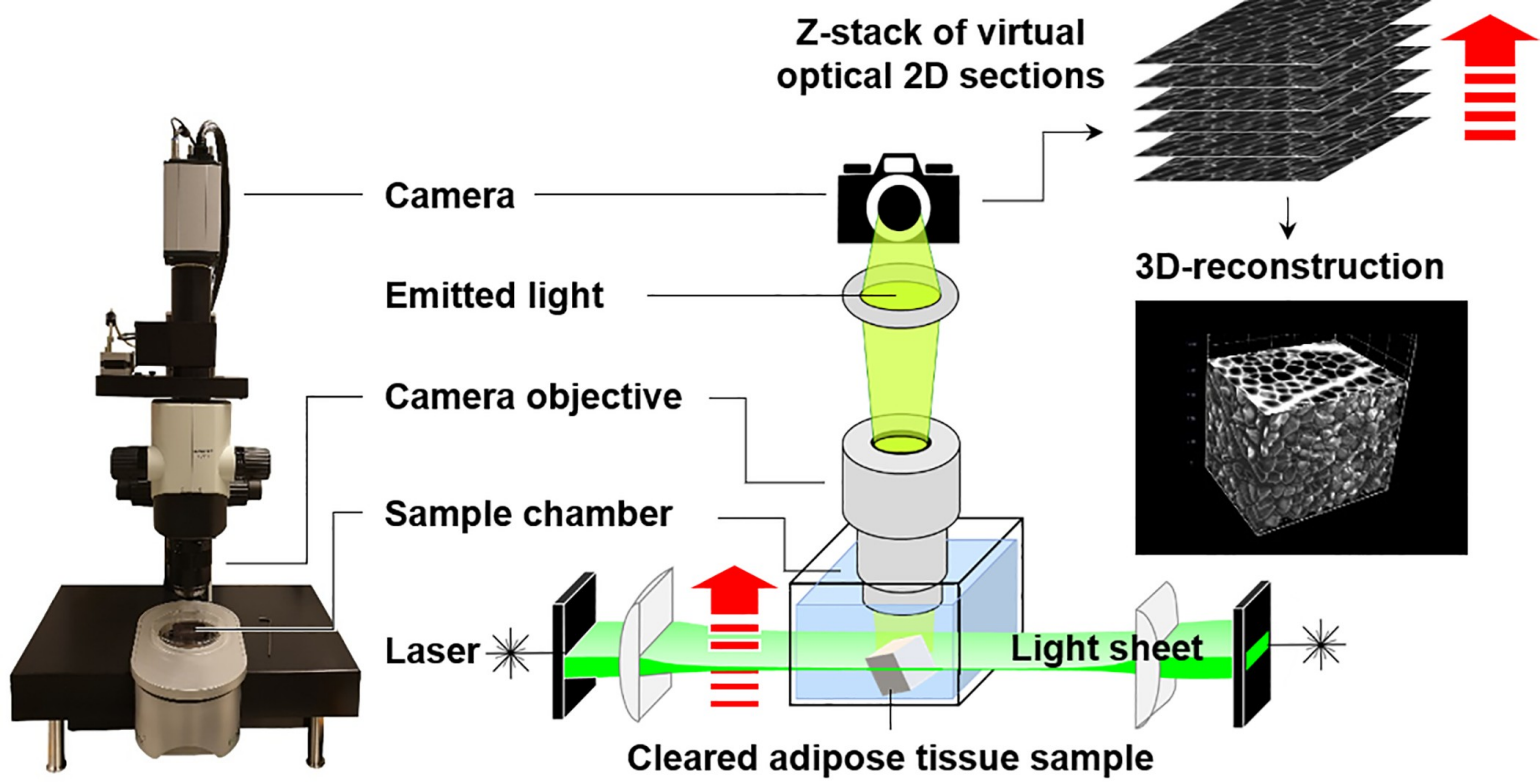
LSFM analysis of cleared adipose tissue samples. An optically cleared adipose tissue sample is placed in the sample chamber of the laser light sheet microscope. A laser light sheet (green) of specific wavelength illuminates the in-focus plane either from one or both sides of the sample. This excites fluorophores within the tissue sample (here: autofluorescence of adipocyte cell membranes) and the emitting light is detected perpendicular to the illumination axis by a digital camera. By moving the sample stepwise (step size: 5 μm) along the vertical axis (red arrow) a series of optical section fluorescence images (z-stack) is acquired, that is used to compute a volume rendered 3D image reconstruction of the adipose tissue sample.

Here, we report the application of 3D light sheet florescence microscopy (LSFM) of optically cleared adipose tissue samples as a simple, fast and elegant approach to characterize the relevant quantitative changes of adipose tissue and adipocyte morphology in different adipose tissue depots of the DIO minipig model. LSFM of optically cleared tissue specimen is a rapidly evolving imaging technique [19–21], allowing for direct 3D imaging of cellular details in tissue samples up to the size of several cm^3^, *e*.*g*., to visualize complex vascularization patterns of organs, tumor samples [22,23], or adipose tissue depots [24]. For LSFM, the tissue specimens are cleared, *i*.*e*., made optically transparent, using a variety of different possible methods and protocols. The cleared samples are then directly (without physically sectioning) imaged in 3D, using a laser light sheet fluorescence microscope. Here, a light sheet of defined laser light wavelength is generated, which illuminates a few microns thin plane of the sample. Fluorescent signals induced within this tissue plane, either by autofluorescence of distinct tissue structures, or by fluorescent marker molecules introduced into the tissue (*e*.*g*., by transgene expression of fluorescent molecules, fluorescence labelled antibodies, lectins or exogenously administered substances), are captured by a camera. As the sample moves through the light sheet, parallel optical fluorescence images are captured, corresponding to the position of the light sheet in the sample. The resulting image stack can be computed to create a digital volume rendered 3D reconstruction of the sample that can be viewed, virtually rotated and sectioned in all three dimensions of space, using appropriate software programs, which may also include tools for digital morphometric image analysis in 3D [20]. **Fig 1** shows a schematic illustration of the LSFM technique for examination of cleared adipose tissue samples. Comprehensive descriptions of the LSFM principle, different clearing methods and applications of LSFM are provided in the pertinent literature [19–21,23,25,26]. In translational obesity research, LSFM has already proven its practicability for 3D imaging of adipose tissue, as well as for the visualization of sympathetic innervation, vascular networks, or inflammatory foci in cleared (murine) adipose tissue samples, using specific fluorescence labelled antibodies or lectins [24,27]. The swift and direct visualization of 3D tissue structures by LSFM, and the direct measurability of morphological features of these structures by digital 3D-image analysis could also efficiently simplify analyses of adipocyte volumes and numbers, which can, so far, only be determined unbiasedly using comparably elaborate and time-consuming quantitative stereological analysis methods. The present study was designed to evaluate the suitability of LSFM-based quantitative morphological analyses to provide accurate (*i*.*e*., precise and unbiased) measures of adipocyte volumes and numbers for characterization of obesity-related alterations of adipocyte growth patterns in different adipose tissue depots of the Göttingen minipig DIO model. To prove the accuracy of the LSFM-analysis results, unbiased quantitative stereological analyses were independently performed on representative samples taken from the same animals and adipose tissue depots. The described methods may serve as a guideline for unbiased quantitative morphological analyses of adipose tissue in future studies involving different translational obesity models and experimental animal species, as well as for examination of human adipose tissue specimens.

## Materials and methods

### Experimental design

Key determinant quantitative morphological parameters of adipocytes (**Table 1**) in subcutaneous (s.c.) and visceral (visc.) adipose tissue depots were determined in obese DIO minipigs and lean control animals, using unbiased quantitative stereological analysis methods, as well as light sheet fluorescence microscopy (LSFM) based analyses of optically cleared adipose tissue samples. All quantitative morphological analyses were performed on representative adipose tissue samples, generated by systematic uniform random (SUR) sampling [8,28]. The volume densities and the total volumes of adipocytes in the adipose tissue were determined in hematoxylin and eosin (HE) stained standard paraffin sections and calculated from the fractional areas of adipocyte cross section profiles within adipose tissue sections and the total volume of the respective adipose tissue depots [8].

**Table 1:** Analyzed quantitative morphological parameters of adipocytes.

Abbreviation	Parameter
**V**_**(ATD)**_	Volume of the adipose tissue depot (ATD)
**V**_**V(AC/ATD)**_	Volume density of adipocytes in an adipose tissue depot
**V**_**(AC,ATD)**_	Total volume of the adipocytes in an adipose tissue depot
**N**_**V(AC/ATD)**_	Numerical volume density of adipocytes in an adipose tissue depot
**N**_**(AC,ATD)**_	Total number of adipocytes in an adipose tissue depot
${\bar{v}}_{\left(\mathrm{AC},\mathrm{ATD}\right)}$	Mean volume of adipocytes in an adipose tissue depot
**V**_**i(AC,ATD)**_	Individual cell volume of an adipocyte in an adipose tissue depot

For LSFM-based quantitative morphological analyses, SUR sampled adipose tissue samples were optically cleared according to an established 3DISCO (3 dimensional imaging of solvent cleared organs) protocol [25] and post-stained with eosin to enhance the autofluorescence signal intensity of (adipocyte) cell membranes for LSFM. 2D optical fluorescence image stacks of the cleared samples acquired by LSFM were computed to digital 3D reconstructions of the samples, using a standard imaging and analysis software. Quantitative morphological analyses of LSFM images for determination of adipocyte numbers and volumes were performed, using the same stereological probes for unbiased sampling and counting of cells as applied in unbiased quantitative stereological analyses. The numerical volume densities of adipocytes within their corresponding adipose tissue depots were determined, using the disector method [8,29]. The total number of adipocytes per adipose tissue depot was calculated from their numerical volume density and the total volume of the respective adipose tissue depot. Individual adipocyte volumes were directly measured in volume rendered 3D reconstructions of the adipose tissue samples, using automated 3D digital image analysis. The mean cellular adipocyte volumes were calculated from the individual volumes and the number of analyzed adipocytes.

The results of the LSFM-based quantitative morphological analyses were verified by unbiased quantitative stereological analysis techniques, using the physical disector method [8,29] with isotropic uniform random (IUR) sections of plastic embedded SUR adipose tissue samples [8,30–32]. The results of both analysis approaches were statistically compared to evaluate the suitability of LSFM of optically cleared adipose tissue samples for quantitative morphological adipocyte analyses.

### Animals

The tissue samples analyzed in the present study were derived from 5 female, ovariectomized, 28 to 30 month old, lean (mean body weight (BW): 46 ± 3 kg) and 6 obese (mean BW: 113 ± 6 kg) Göttingen minipigs. These animals had been euthanized and necropsied in the course of a previously published study on the DIO minipig model [6]. All animal experiments were conducted according to the German Animal Welfare Act and the ARRIVE guidelines and Directive 2010/63/EU, and with approval of the ethics committee of the Government of Upper Bavaria and its permission (permission number: AZ 55.2-1-54-2532-60-2015). Pigs were anesthetized by intramuscular injection of ketamine (Ursotamin, Serumwerke Bernburg, 20 mg/kg BW) and azaperone (Stresnil, Elanco, 2 mg/kg BW) followed by intravenous application of ketamine and xylazine (Xylazin 2%, Serumwerk Bernburg). The animals were then euthanized under anesthesia by intravenous injection of pentobarbital (Release, WDT, at least 450 mg/10 kg BW) and immediately subjected to necropsy.

### Volume determination of adipose tissue depots

Two different (white) adipose tissue depots within previously defined anatomical borders [6] were examined, the subcutaneous (s.c.) adipose tissue depot of the back and the visceral (visc.) adipose tissue. The s.c. adipose tissue depot was defined as the subcutaneous adipose tissue covering the dorsal aspect of the back from the first to the last lumbar vertebra, ventrally limited by the plane of the transverse processes of the lumbar vertebrae. The visceral adipose tissue was defined as the subperitoneal adipose tissue attached to the abdominal wall, excluding the omental-, and mesenteric-visceral adipose tissue. At necropsy, these adipose tissue depots were separated mechanically and weighed to the nearest gram. The density of the adipose tissue was determined using the submersion technique, as previously described [6,30,31], and accounted for ρ = 0.9 ± 0.02 g/cm^3^ on the average (with no significant differences between different adipose tissue depots in lean and obese pigs). The volumes of the different adipose tissue depots were calculated from their weights and density [6,30,31].

### Systematic uniform random (SUR) sampling of adipose tissue specimen

For unbiased quantitative stereological analyses, as well as for LSFM-based quantitative morphological analyses, each 6 representative tissue specimens of approximately 1 cm x 1 cm x 1 cm were systematically uniformly randomly (SUR) sampled from the s.c. and the visc. adipose tissue depots of each pig (**S1 Fig**), as previously described [6,30]. In the visceral adipose tissue depot, samples were only taken from areas without macroscopically evident inflammatory alterations. The excised tissue samples were fixed in neutrally buffered 4% formaldehyde solution for >12 hours.

### Determination of the volume densities and of the total volumes of adipocytes per adipose tissue depot

The formalin-fixed adipose tissue samples destined for unbiased quantitative stereological analyses were bisected. One half of each sample (*i*.*e*., 6 s.c. and 6 visc. adipose tissue SUR samples per case) were routinely embedded in paraffin in arbitrary orientation, sectioned and stained with hematoxylin and eosin (HE). The volume density of adipocytes in an adipose tissue depot (V_V(AC/ATD)_) was determined, using the Visiomorph image analysis system with Newcast software (Visiopharm A/S, Denmark). V_V(AC/ATD)_ was calculated as the fractional area of section profiles of adipocytes and the corresponding adipose tissue in 29 ± 5 SUR sampled fields of view at 100x microscopic magnification, using the point counting method (**S2 Fig**), as described earlier [8,31,33]. Per case, 92 ± 15 points were counted, on the average. The total volume of adipocytes in an adipose tissue depot (V_(AC,ATD)_) was calculated as the product of V_V(AC/ATD)_ and the total volume of the adipose tissue depot (V_(ATD)_).

### Sample processing for LSFM-based quantitative morphological analyses

**Fig 2:**
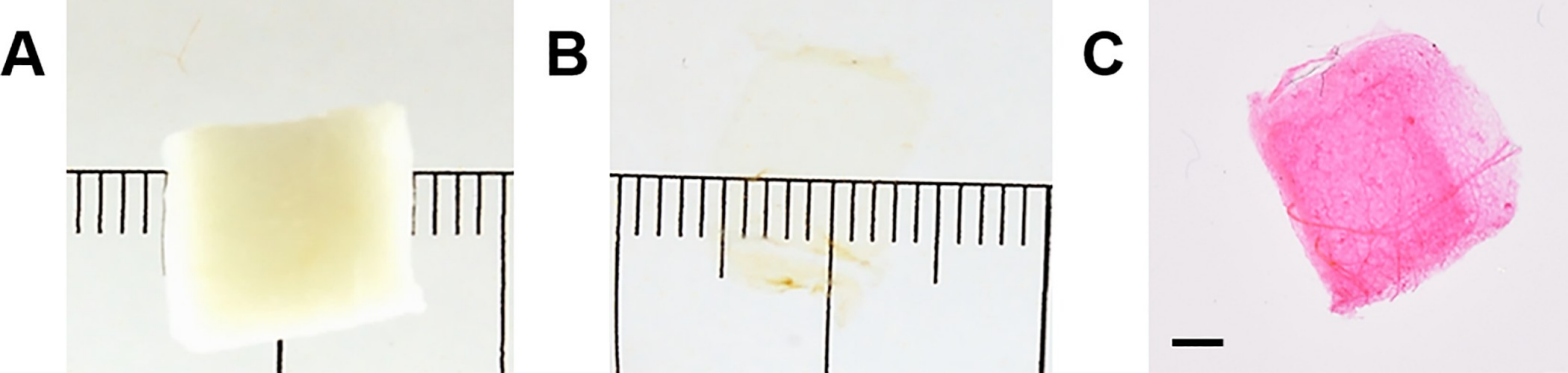
Optical clearing and eosin staining of cleared adipose tissue samples. **A.** SUR sampled, formalin-fixed adipose tissue sample. **B.** Adipose tissue sample from A after 3DISCO clearing. Note the transparency and the reduced size of the cleared tissue sample, as compared to **A**. **C.** Eosin stained, cleared adipose tissue sample. Bar = 1 mm.

**Fig 3:**
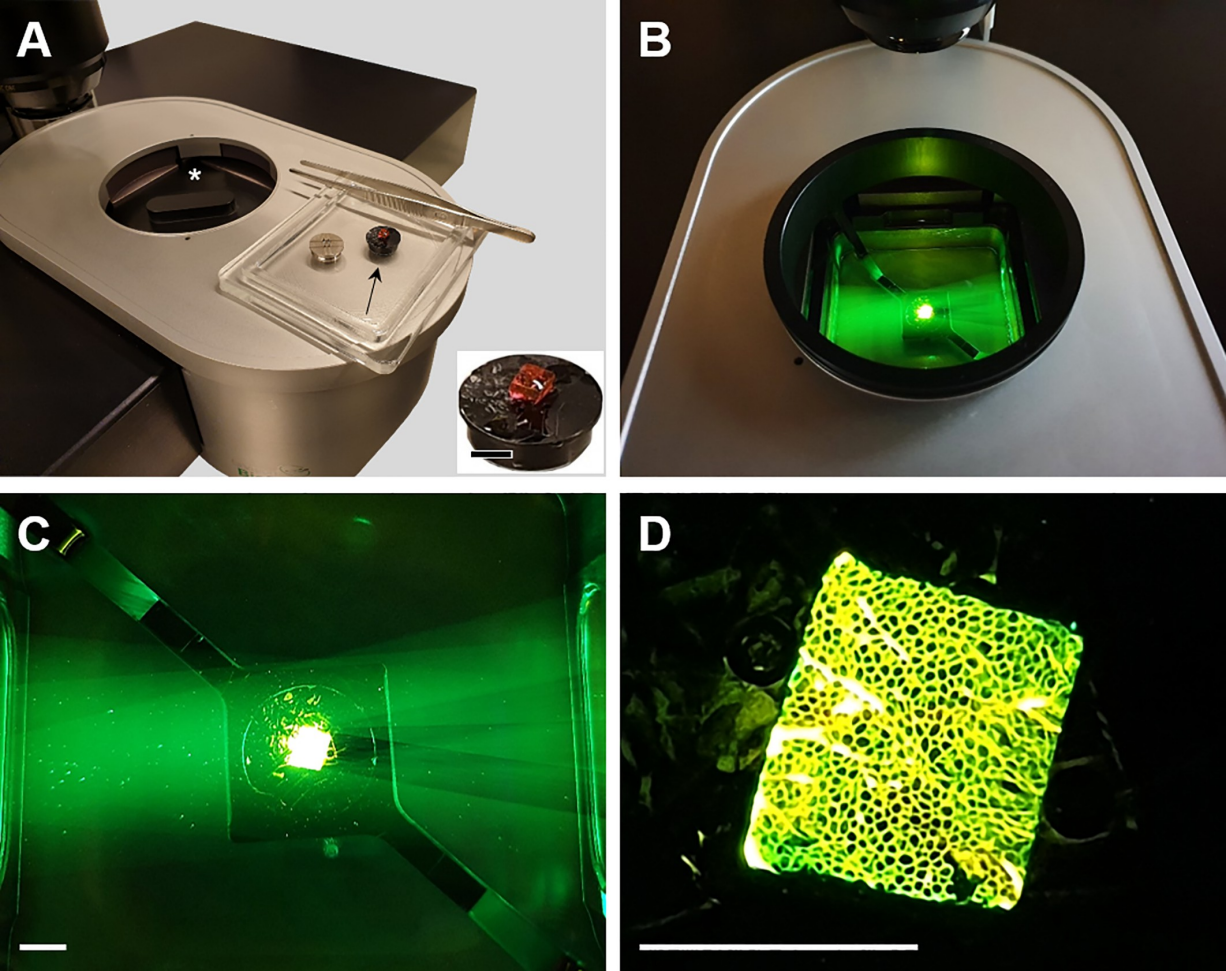
LSFM analysis of 3DISCO cleared, eosin-stained adipose tissue samples. **A.** Bottom part of the laser light sheet microscope (UltraMicroscope 2, LaVision Biotec). A cleared and eosin-stained adipose tissue sample is mounted on a specimen holder (black arrow). The asterisk marks the sample chamber (closed with a lid). Inset: Detail enlargement of the 3DISCO cleared, eosin-stained adipose tissue sample. **B.** Adipose tissue sample illuminated by a laser light sheet (green light, 520/40 nm) in the sample chamber. **C.** Detail enlargement of **B**. Note the green laser light sheet and the green-yellow autofluorescence of the sample. **D.** Macroscopic picture of the laser illuminated adipose tissue sample. Single adipocytes are already grossly discernible.

SUR sampled, formalin-fixed adipose tissue specimens were optically cleared (**Fig 2**), using a slightly modified version of the established 3DISCO protocol [25], with chemicals purchased from Sigma Aldrich, Germany. After washing off the fixation solution in tab water for 2 hours, the samples were processed through series of clearing solutions: 50% tetrahydrofuran (THF, 1h), 50% THF (12h), 70% THF (8h), 80% THF (12h), 100% THF (12h), 100% THF (1h), 100% dichloromethane (DCM, 30 min. until the tissue sample sinks), benzyl alcohol benzyl benzoate (BABB, 10 min.), BABB (20 min.), BABB (30 min). To avoid compression of samples, the samples were placed in individual, sufficiently large containers and cleared in large volumes of the respective clearing solvents (*i*.*e*., “free-swimming” samples; tissue-to-solvent ratio approximately 1:20). To increase the autofluorescence signal intensity of adipocyte cell membranes (**Figs 2C and 3**), the cleared tissue samples were then stained with eosin solution (ST Infinity H&E staining system, ref. 3801098, Leica, Germany) for 45 minutes, after rehydrating the samples in a series of 100% isopropyl alcohol (30 min.), and 100%, 96%, 90%, 80%, 70%, 50% ethanol and tab water (each for 30 min.). The stained adipose tissue samples were rinsed in tab water until the added water remained unstained, and then dehydrated in the same alcohol series applied for rehydration, transferred to BABB, and stored in in BABB in the dark at 4–8°C until further examination.

The extent of tissue shrinkage associated with the clearing and staining process was calculated from the individual adipose tissue sample volumes of 16 s.c. and visc. adipose tissue samples of lean and obese pigs, determined before and after the clearing/staining procedure, using the submersion technique [30,31,34] with 4% formaldehyde solution (ρ = 1.0165 g/cm^3^) or BABB (ρ = 1.0969 g/cm^3^) as submersion liquids (at 20°C).

### LSFM and quantitative morphological analyses

**Fig 4:**
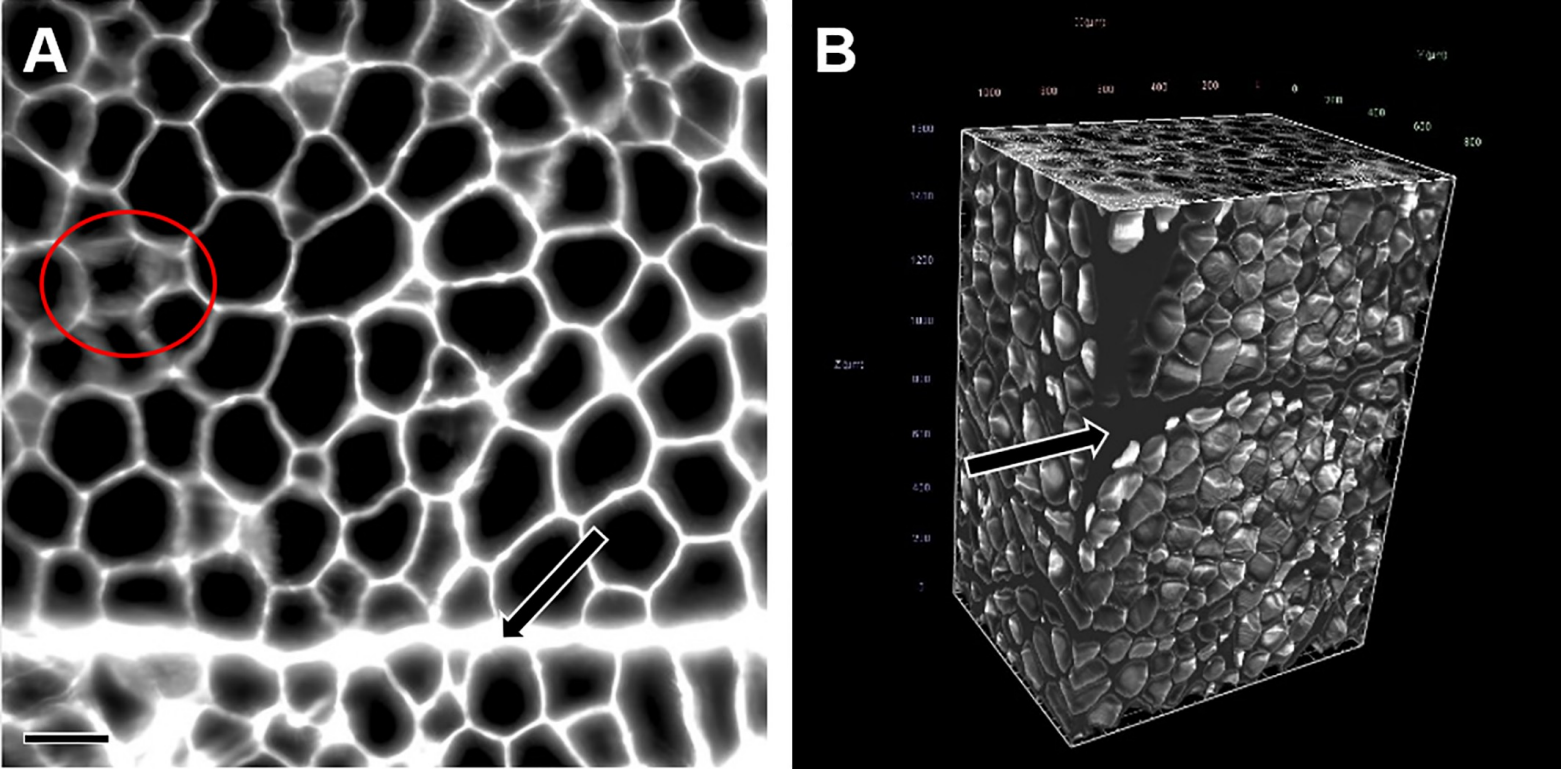
2D LSFM section image and digital 3D reconstruction of an adipose tissue sample. **A.** 2D LSFM autofluorescence image from an optical section image stack acquired in a 3DISCO cleared, eosin stained s.c. adipose tissue sample. Autofluorescence signals of adipocyte cell membranes acquired at 585/40 nm (emission range) wavelength (excitation range: 520/40 nm) and are shown in white color. Areas of indistinctly broadened cell membranes (encircled in red) represent tangential sections of cell membranes (adipocyte “tops” or adipocyte “bottoms”). **B.** Digital 3D reconstruction of the s.c. adipose tissue sample, computed from a z-stack of 322 optical section plane images with a step size of 5 μm, containing the image shown in **A**. The 3D reconstruction is freely rotatable and can be virtually sectioned in all directions of space (compare to **S1 Video**). Note the presence of strands of connective tissue traversing through the s.c. adipose tissue sample (arrows in **A** and **B**). Bar = 100 μm.

**Fig 5:**
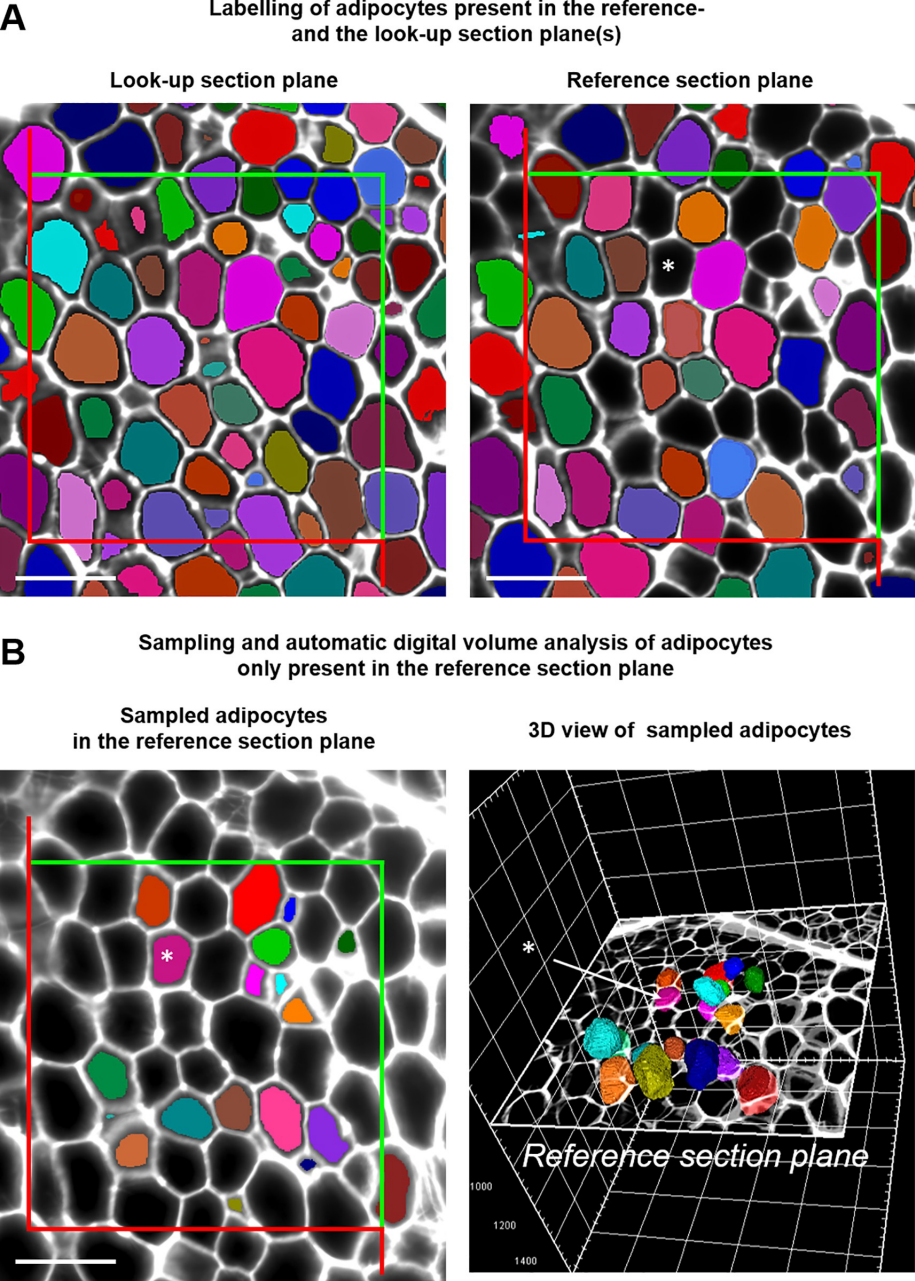
Unbiased number weighted sampling of adipocytes in 3D LSFM image reconstructions of optically cleared adipose tissue samples with the disector for determination of adipocyte numbers and individual adipocyte volumes. **A.** Randomly sampled 2D optical LSFM autofluorescence image planes (look-up- and reference-section plane) with a defined distance (50 μm). Corresponding fields of view in the look-up section plane and the reference section plane are superimposed with unbiased counting frames. All adipocyte cross section profiles present in the look-up section are marked with the “*Magic Wand*” tool included in the arivis Vision4D imaging and analysis software and labelled with individual colors. Adipocytes which also occur in the reference section are automatically displayed in the same colors. Section profiles of adipocytes which are sectioned by the reference section plane, but not by the look-up section plane remain unlabelled (black). For illustration purposes, one cross section of an adipocyte that is sectioned by the reference section plane but not by the look-up section plane is highlighted by an asterisk. Adipocytes which are only sectioned by the reference section plane but not by the look-up section plane are than sampled, using unbiased counting frames, *i*.*e*., cells are only sampled/counted, if their section profiles are either entirely located within the sampling frame, or if they touch one of the “inclusion” lines (green lines) without touching one of the “exclusion” lines (red). **B**. Disector sampled adipocytes in the 2D reference section plane (left image) and in the 3D LSFM image reconstruction of the adipose tissue sample, virtually sectioned at the level of the disector reference section (right image). The sampled adipocytes are labelled with the “*Magic Wand*” tool, which also calculates the individual adipocyte cell volumes. Individual adipocytes and their corresponding section profiles are shown in corresponding, individual colors. The asterisks mark the same adipocyte as in A. Bars = 200 μm.

For LSFM analysis (**Figs 2–5, S1 Video**), the 3DISCO cleared, eosin-stained adipose tissue samples were examined with an UltraMicroscope II—zoom box configuration (LaVision BioTec GmbH, Germany) with dual side illumination and triple sheet optics on each side generating laser light sheets perpendicular to the direction of detection. The microscope was equipped with a SuperK EXTREME EXW12 white laser (NTK Photonics, Germany) with a wavelength range from 465 to 2400 nm, a 2x objective lens (Olympus MVPLAPO 2X/0.5 NA) combined with an adjusted, custom-made dipping cap (LaVisionBiotec) optimized for liquids with a refractive index of 1.56 ± 0.1, an Olympus MVX-10 zoom body (Olympus, Hamburg, Germany) providing magnification steps from 0.63x to 6.3x, and a Andor Zyla 4.2 Plus sCMOS camera (Oxford Instruments GmbH, Germany). BABB was used as medium. Z-stacks of fluorescence images of 5 μm optical thickness were acquired at 520/40 nm (excitation range) and 585/40 nm (emission range) for detection of autofluorescence (**Fig 4**). With the used objective lens and the applied analysis settings, also a lateral resolution of approximately 5 μm was achieved. 3D volumetric sample reconstruction images (**Fig 4, S1 Video**) were computed, using ImSpector Pro^64^ (vers. 5.1.328, LaVision Biotec GmbH, Germany), and arivis Vision4D (vers. 3.0, arivis, Germany) software tools.

For control of the LSFM-image (pixel/voxel-size) scaling and to monitor the realistic rendering of object shapes and volumes in digital 3D-LSFM reconstructions, fluorescently labelled, spherical microbeads (PS-FluoRot 100, batch: PS-FluoRot-Fi274, microParticles GmbH, Germany) with a defined diameter of 99.9 ± 1.8 μm (*i*.*e*., similar to the size of adipocytes) were used as control objects. These polystyrene beads are shape- and volume-stable (non-shrinking), resistant to organic and aqueous solvents, have a smooth, non-porous surface, display a refractive index of 1.59 (*i*.*e*., similar to the refractive index of BABB (1.56)) and exhibit a red fluorescence at Ex/Em 530nm/607nm. The mean physical (“true”) volume of the microbeads (522 ± 28 x10^3^ μm^3^) was calculated (V = (4/3)_*_π_*_r^3^) from their mean diameter and standard deviation (as indicated by the manufacturer and verified by microscopic measurements of 50 randomly selected beads, **S4A Fig**). For LSFM-imaging, approximately 100 microbeads were immobilized by embedding in a ~0.5 cm^3^ cube of agar (Agar-Agar, Kobe I, Carl Roth GmbH & Co KG, Germany). The agar block was fixed in 4% neutrally buffered formaldehyde solution (12 hours) and subsequently processed according to the 3DISCO-clearing protocol described above. The microbead control sample was then imaged and analysed, using the same LSFM-instrument-, image-acquisition-, and analysis-settings, as for analysis of adipose tissue samples. Additionally, the correct calibration of the z-stage drive of the LSFM-instrument was confirmed in regular intervals, using a digital micrometer (RS Components GmbH, Germany). The diameters and volumes of microbeads were measured in the digital LSFM z-stack images and corresponding 3D reconstructions (**S4B–S4E Fig**) and compared to the “true” physical bead-diameters and -volumes.

For analysis of adipocyte numbers in the adipose tissue depots, the mean adipocyte volumes (v_(AC,ATD)_) and the individual adipocyte volumes (V_i(AC,ATD)_), adipocytes were unbiasedly sampled using the disector method [8,29,35]: from each 2D autofluorescence LSFM image stack, two technically impeccable optical section plane images (a reference section and a look up section) with a distance of 50 μm (*i*.*e*., 10 section planes apart) were randomly sampled from the middle third of the image stack. Using the “split screen” function of the arivis Vision4D software, both optical section planes were displayed side by side on the same screen. In both optical section planes, corresponding fields of view were SUR sampled at appropriate magnification. Adipocytes sectioned by the reference section, but absent in the look-up section were identified, using the “*Magic Wand*” tool of the arivis Vision4D software (analysis settings: dark objects, tolerance: 10000), as illustrated in **Fig 5**. Using unbiased counting frames [7,8,29,36,37] superimposed on the digital images of the corresponding fields of view, 116 ± 23 adipocytes were sampled and counted per adipose tissue depot, on the average. The “*Magic Wand*” tool was also used to directly label individual adipocytes in the volume rendered 3D image reconstruction of the adipose tissue sample, and to measure the individual cell volumes of adipocytes unbiasedly sampled with the disector (based on the number of voxels within the unstained centre of the cell that is completely surrounded by a continuously stained cell membrane). The measured individual adipocyte cell volume values were subsequently corrected for the shrinkage associated with the 3DISCO clearing and eosin staining procedure of the tissue samples. The mean adipocyte volume (v_(AC,ATD)_) was calculated as the mean of the (shrinkage corrected) individual adipocyte volumes measured in all tissue samples from one adipose tissue depot. The total numbers of adipocytes (N_(AC,ATD)_) in the s.c. and visc. adipose tissue depots were calculated from total volume of adipocytes and the corresponding mean adipocyte volume(s).

### Verification of LSFM-based quantitative morphological analysis results by unbiased quantitative stereological analyses

For verification of LSFM-based quantitative morphological analysis results, each twelve SUR sampled, formalin-fixed, halved, subcutaneous and visceral adipose tissue samples of lean and obese pigs were randomly sampled and processed for estimation of cell numbers and mean volumes by unbiased quantitative stereological analyses. The adipose tissue samples were processed to generate isotropic uniform random (IUR) sample section planes, using the orientator method, as described previously [6,30–32]. For shape stability and better visual contrast, the fixed adipose tissue samples were encased in ink-blackened agar [30] to avoid mechanical deformation/compression of soft adipose tissue samples during the further processing steps (**S3 Fig**). The IUR samples were then embedded in glycolmethacrylate/methylmethacrylate (GMA/MMA) plastic embedding medium [38]. From each GMA/MMA embedded tissue block, a series of at least nine consecutive serial sections with a nominal section thickness of 1.00 μm was cut. The factual section thicknesses were measured by spectral reflectometry, as previously described in detail [35] and accounted for 1.34 ± 0.28 μm on the average. The sections were then stained with HE.

**Fig 6:**
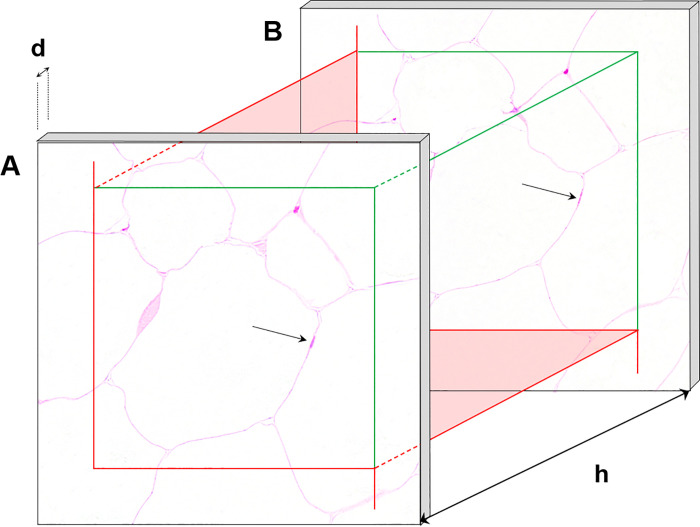
Illustration of the physical disector method for unbiased sampling and counting of adipocytes (nuclei). A physical disector consists of two parallel, corresponding tissue sections (**A**, **B**) of known distance, randomly sampled from a series of consecutive sections. The distance of both sections (*i*.*e*., the disector height, h) is defined by the thickness of sections (d), and by the number of sections between them. The aligned (SUR sampled) fields of view are superimposed with unbiased counting frames of known area with exclusion (red) and inclusion lines (green). Thus, a specified tissue volume is defined between both section planes, in which particles, such as cells or cell nuclei, can be unbiasedly sampled and counted independent of their shape, size, or orientation. For analysis of numerical volume densities of (uninuclear) cells, usually cell nuclei are sampled with the physical disector, since they can easily be rediscovered in disector sections with a disector height of approximately one third of the mean minimal nucleus diameter. Using the unbiased counting frames, particles are counted, if they are present within the first (reference) section (**A**) but absent in the second (look-up) section (**B**).

The tissue shrinkage associated with the embedding in GMA/MMA was calculated from the quotient of the IUR section plane area of the tissue sample prior to GMA/MMA embedding and the tissue section profile area of the first (complete) section, as described previously (**S3 Fig**) [31]. On the average, the GMA/MMA embedding related volume shrinkage of adipose tissue samples accounted for 21 ± 0.001%, corresponding to a linear tissue shrinkage factor f_s_ of 0.93 ± 0.0006. The numerical volume density of adipocytes per adipose tissue depot (N_V(AC/ATD)_) was determined, using the physical disector principle (**Fig 6**), as described earlier [8,29,35]. From each series of consecutive (nominally) 1 μm thick GMA/MMA sections, two neighboured sections were randomly sampled. In the first section (reference section) 15 ± 8 fields of view were SUR sampled at 200x magnification and superimposed with unbiased counting frames of 300 μm x 300 μm edge length [7,8,29,36,37]. The corresponding section areas in the second section (look-up section) were located, aligned to the reference section, and also superimposed with unbiased counting frames. Using the unbiased counting frames, adipocyte cell nuclei (not adipocytes) were counted (Q^-^), if their section profiles were present in the reference section but absent in the look-up section (**Fig 6**). The counting process was then repeated with interchanged roles of the reference- and look-up sections, thus doubling the efficiency of the counting process. Per adipose tissue depot, 62 ± 12 adipocytes (nuclei) were counted in 242 ± 124 fields of view in 16 ± 3 analyzed pairs of disector sections, on the average. N_V(AC/ATD)_ was then calculated as described in **Eq 1**.

The corresponding coefficients of error (CE) of the numerical volume density estimates, considering the variances originating from the sampling within sections, were calculated as described previously [39] and accounted for 0.1 ±0.01 on the average. The total number of adipocytes in an adipose tissue depot (N_(AC,ATD)_) was calculated as the product of N_V(AC/ATD)_ and V_(ATD)_. The mean volume of adipocytes in an adipose tissue depot (v_(AC,ATD)_) was calculated as the quotient of V_V(AC/ATD)_ / N_V(AC/ATD)_.

### Eq 1. Numerical volume density of adipocytes in an adipose tissue depot

$$N_{V(AC/ATD)}=\left(\frac{\sum Q^{-}}{h*\sum A_{(ATD)}}\right)*f_{s}^{3}$$

N_V(AC/ATD)_ Numerical volume density of adipocytes (AC) in an adipose tissue depot (ATD).

∑Q^-^ Cumulative number of all counted adipocyte cell nuclei (Q^-^) in all disectors examined per adipose tissue depot.

h Disector height, *i*.*e*., the distance between the reference and the look-up section.

∑A_(ATD)_ Cumulative adipose tissue section area within the unbiased counting frames in all SUR sampled reference section fields of view.

h x ∑A_(ATD)_ Cumulative volume of all disectors examined per adipose tissue depot.

f_s_ Linear tissue shrinkage factor (0.93 for GMA/MMA embedded porcine adipose tissue, and 0.92 for 3DISCO cleared and HE stained porcine adipose tissue).

### Histomorphometric measurement of adipocyte cross section profile parameters

In order to also provide histomorphometric measurements of “commonly used” 2D adipocyte section profile parameters, the mean adipocyte section profile-areas, and -(maximal) diameters, as well as the adipocyte section profile-numbers per section area (*i*.*e*., the numerical area densities of adipocyte section profiles) were determined in HE-stained IUR-cryo-sections [8,30,32,40] prepared from each three systematically randomly sampled, subcutaneous and visceral adipose tissue samples from 3 lean-, and 3 obese pigs. Per case, an average of 1054 ± 405 adipocyte section profiles were randomly sampled with unbiased counting frames and analyzed using a standard automated image analysis software (Definiens, Germany).

### Statistical analyses

Data are presented as means ± standard deviations per group (lean and obese minipigs) and adipose tissue depot (s.c. and visc.). Distribution and variance homogeneity analyses and statistical comparisons between different groups were performed, using GraphPad Prism (version 5.04, GraphPad Software, USA) and Microsoft Excel (Microsoft, USA), with appropriate statistical tests, as indicated.

## Results

### Total adipocyte volumes in subcutaneous and visceral body fat depots of lean and obese minipigs

The volume densities of adipocytes in the subcutaneous adipose tissue depot (V_V/(AC/ATD)_) did not significantly differ in obese (0.91 ± 0.04%) and lean (0.87 ± 0.04%) minipigs, whereas the volume densities of adipocytes in the visceral adipose tissue depot were significantly increased in obese (0.93 ± 0.02%) *vs*. lean (0.89 ± 0.01%) minipigs.

**Fig 7:**
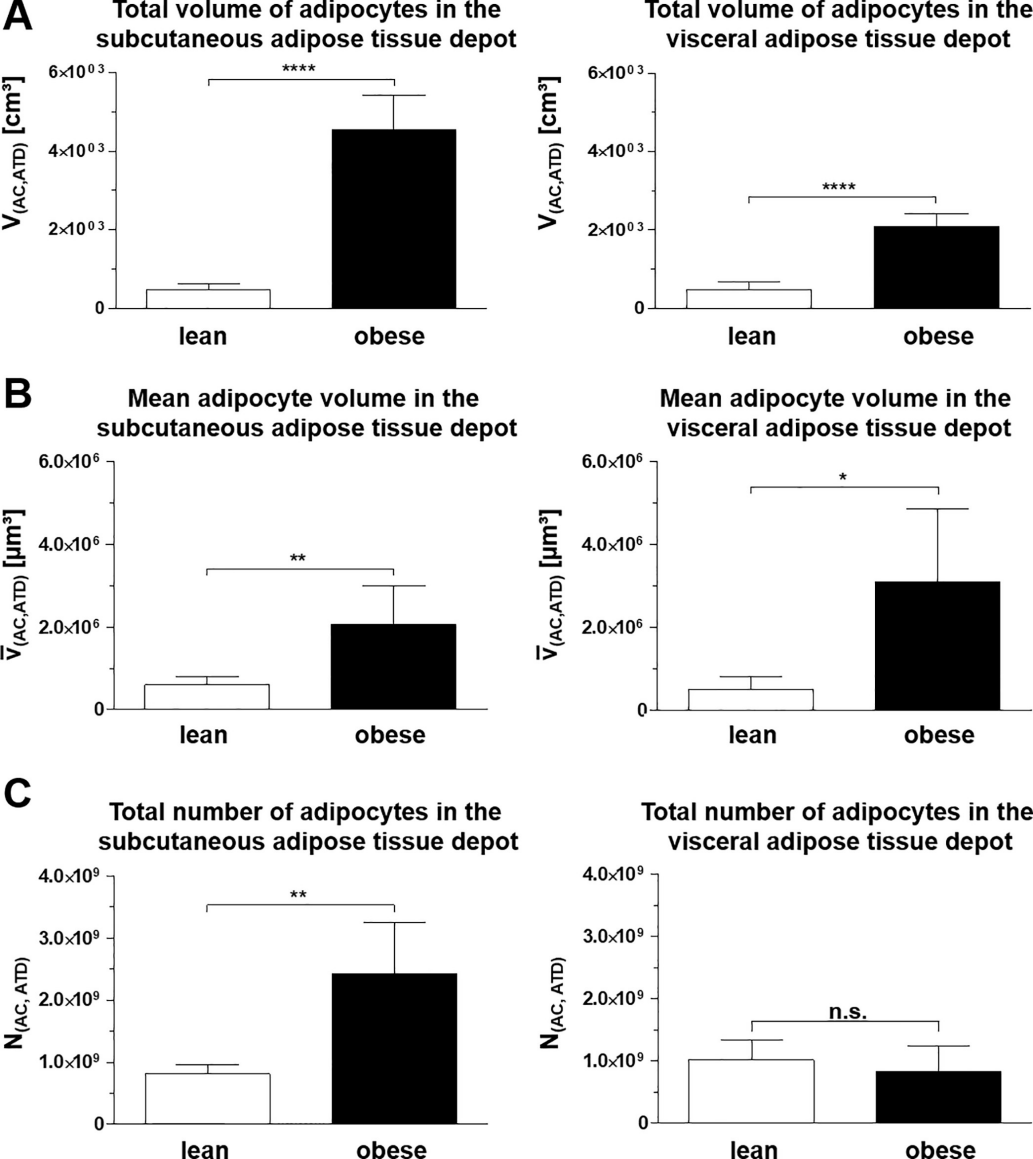
Key determinant quantitative morphological adipocyte parameters in s.c. and visc. adipose tissue depots of lean (n = 5) and obese (n = 6) minipigs. **A.** The total volumes of adipocytes in the s.c. and the visc. adipose tissue depots were determined from the volume density of adipocytes in the adipose tissue and the total volume of the respective adipose tissue depot. **B, C.** The mean volumes and the total numbers of adipocytes in an adipose tissue depot were obtained by LSFM-based quantitative morphological analyses. Data are means ± standard deviations. t-test. *: p < 0.05; **: p < 0.01; n.s.: not significant (p > 0.05).

In both adipose tissue depots, the total adipocyte volume was significantly increased in obese *vs*. lean minipigs, by >860% (s.c.), and by >340% (visc.), respectively (**Fig 7A**).

### LSFM-based quantitative morphological analyses of adipocyte volumes and numbers

LSFM of 3DISCO cleared, eosin-stained adipose tissue samples allowed for an unambiguous autofluorescence detection of continuous adipocyte cell borders and generation of precise 3D sample reconstructions from optical LSFM image stacks, with identification of single adipocytes (**Figs 4 and 5**, **S1 Video**). The applied clearing/staining procedure was associated with a volume shrinkage of the tissue samples of 21 ± 5.4% (corresponding to a linear tissue shrinkage factor of f_s_ = 0.92) on the average (without significant differences between lean and obese minipigs or s.c. and visc. adipose tissue samples). Using the arivis Vision4D vers. 3.0 imaging and analysis program for generation, viewing, volume rendering, and analysis of 3D tissue sample reconstructions enabled an easy and swift acquisition of individual volumes of adipocytes, unbiasedly sampled with a disector probe.

The correct LSFM-image (pixel/voxel-size) scaling and rendering of object shapes and volumes in digital 3D-LSFM reconstructions was confirmed by imaging and volume analysis of fluorescent microbeads with a defined size comparable to that of adipocytes. In their digital 3D-image reconstructions, the beads appeared as spheres (*i*.*e*., retainment of shape, **S4 Fig**) and the bead volumes determined in their digital 3D-images (534 ± 14 x10^3^ μm^3^) precisely matched with the “true” bead volumes calculated from their physical diameters (522 ± 28 μm^3^), with a mean deviation of under 5% (p > 0.3). Compared to 2D section based unbiased quantitative stereological analyses, LSFM-based quantitative morphological analyses were associated with considerably lower time- and work-expenditure. This difference was primarily due to the considerably faster sample clearing process and LSFM imaging procedure, as compared to the generation and analysis of histological (serial) sections of plastic embedded tissue samples.

### Obesity associated changes of adipocyte numbers and mean volumes in subcutaneous and visceral body fat depots identified by LSFM-based quantitative morphological analyses

In both lean and obese minipigs, respectively, the mean cellular volumes of adipocytes (v_(AC,ATD)_) in the subcutaneous adipose tissue depots were not significantly different from the mean volumes of adipocytes in the corresponding visceral adipose tissue depots of the same animals (paired t-tests; p > 0.3).

The adiposity associated increase of the total volumes of both the subcutaneous and the visceral adipose tissue depots in obese *vs*. lean minipigs was consistently associated with significant increases of the mean cellular volumes of adipocytes. On the average, the obesity associated increase of the mean volume of adipocytes in the subcutaneous adipose tissue depot of lean *vs*. obese animals accounted for 361%, and 406% in the visceral adipose tissue depot (**Fig 7B**).

In the subcutaneous adipose tissue depot, the total number of adipocytes per adipose tissue depot (N_(AC,ATD)_) was also significantly increased in obese *vs*. lean minipigs, accounting for an almost triplication (270% increase) of adipocyte numbers. In the visceral adipose tissue depot, however, the obesity associated increase of the mean cellular volumes of adipocytes in obese *vs*. lean minipigs was not associated with a significant (p = 0.44) increase of the total numbers of adipocytes (**Fig 7C**).

**Fig 8:**
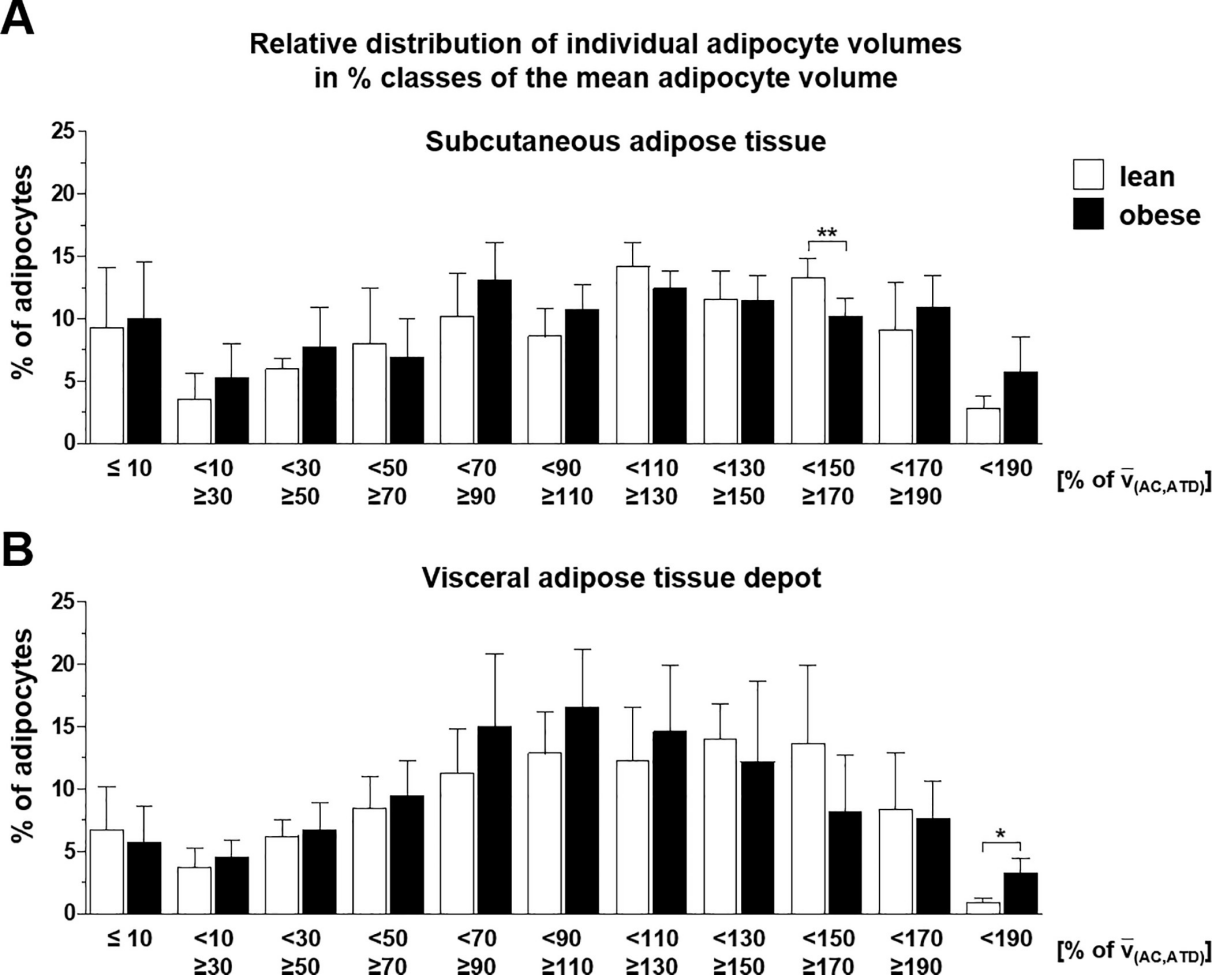
Relative distribution of individual adipocyte volumes in % classes of the mean adipocyte volume in the subcutaneous adipose tissue depot (A) and the visceral adipose tissue depot (B) of lean (n = 5) and obese (n = 6) minipigs (obtained by LSFM-based analyses of optically cleared adipose tissue samples). The bars represent the percentual numbers of adipocytes with individual volumes ranging in 20% classes of the mean adipocyte volume. Data are means ± standard deviations. t-test. *: p < 0.05, **: p < 0.01.

The measured individual adipocyte volumes (V_i(AC,ATD)_) ranged over three decimals from 0.013 x 10^6^ μm^3^ to 15.9 x 10^6^ μm^3^, with the largest adipocytes being present in the visceral adipose tissue of obese minipigs. Regardless of the increased mean cellular adipocyte volumes in obese *vs*. lean minipigs, the individual adipocyte volumes in both s.c. and visc. adipose tissue samples of lean and obese minipigs were normally distributed (**Fig 8**).

### Verification of LSFM-based quantitative morphological analysis results by unbiased quantitative stereological analyses

The results of the LSFM-based analyses of the absolute numbers of adipocytes in the different adipose tissue depots (N_(AC,ATD)_), and of the mean adipocyte volumes in optically cleared s.c. and visc. adipose tissue samples of lean and of obese minipigs did not significantly differ from the corresponding values obtained by unbiased quantitative stereological analyses (paired t-test; p > 0.4). Indeed, the mean percentual deviation of the results obtained by both analysis methods was only 1.04 ± 3.0% for the total numbers of adipocytes, and 1.14 ± 3.1% for the mean volumes of adipocytes in the s.c. and the visc. adipose tissue depots in lean and obese minipigs.

### Histomorphometric measurement of adipocyte cross section profile parameters

The mean adipocyte section profile -areas, and -(maximal) diameters in sections of s.c. and visc. adipose tissue depots of obese minipigs (s.c.: 16545 ± 3174 μm^2^, and 179 ± 17 μm; visc.: 24196 ± 4796 μm^2^, and 207 ± 21 μm) were significantly (p > 0.05) increased (by > 200%, respectively by > 150%), as compared to lean control animals (s.c.: 8088 ± 1476 μm^2^, and 122 ± 9 μm; visc.: 10548 ± 3031 μm^2^, and 139 ± 20 μm). In contrast, the numerical area densities of adipocyte section profiles were significantly higher (> 50%) in lean (s.c.: 1156 ± 189 n/10^6^ μm^2^; visc.: 769 ± 90 n/10^6^ μm^2^) than in obese minipigs (s.c.: 557 ± 91 n/10^6^ μm^2^; visc.: 386 ± 83 n/10^6^ μm^2^).

## Discussion

### Discussion of study aims and the general experimental design

The aims of the present study were to evaluate the suitability of LSFM-based quantitative morphological analyses to provide precise and unbiased measures of adipocyte volumes and numbers to characterize obesity-related, fat depot specific alterations of adipocyte growth patterns in the large animal model (LAM) of diet induced obesity (DIO) in Göttingen minipigs [6]. Methodologically, the use of an LSFM-based approach for quantitative volume- and number-analyses of adipocytes was motivated by the principal advantages of LSFM-analyses, allowing for a quick and straightforward 3D-visualization and morphological analysis of tissue elements in optically cleared, intact (adipose) tissue samples [24,27,41]. Provided the reliability and accuracy of obtained measurement results, LSFM-based analyses thus hold a great potential to efficiently examine relevant quantitative (and qualitative) histomorphological parameters of adipocytes with less effort and analytical travail than conventional quantitative morphological analysis methods that depend on 2D histological sections.

The experimental design of the study was thus conceived to warrant an objective evaluation of the accuracy of LSFM-based quantitative morphological measurement data of adipocyte volumes and numbers. For this, LSFM-measurement data were compared to the results of adipocyte volume and number analyses obtained by unbiased quantitative stereological analysis methods, representing the gold-standard of quantitative histomorphological analyses in biological tissue samples.

To warrant the comparability and unbiasedness of the results obtained by both analysis approaches, representative adipose tissue samples (of identical animals) were generated by systematic uniform random sampling [8,28,30,31,42] and either subjected to LSFM-analysis, or processed for unbiased quantitative stereological analysis. Both analyses approaches used the disector method [8,29,35] for unbiased sampling and analysis of adipocytes, ensuring that the selection of analyzed adipocytes within the examined tissue samples were not biased by the size, shape, and orientation of the adipocytes.

Factors confounding the accuracy of quantitative morphological analysis results in both approaches, such as the embedding-, respectively clearing-related shrinkage of tissue samples [43], incorrect and/or variable factual thicknesses of the analyzed histological sections [35], and potential LSFM-imaging artifacts (leading to a distortion of adipocyte shapes and volumes in digital 3D image reconstructions generated from LSFM-image z-stacks) [44] were precisely determined and corrected, or, respectively, minimized by using adequate technical equipment and defined test-particles as shape/volume-controls.

### Discussion of the LAM-DIO model and relevant quantitative histomorphological adipocyte parameters

In the present study, the established LAM of DIO in Göttingen minipigs was used to objectively quantify and characterize obesity related alterations of growth patterns of (unilocular) adipocytes in different adipose tissue depots [6]. Animals of this DIO model reproducibly develop pathogenetically relevant metabolic derangements and histological alterations which are also observed in the human metabolic syndrome (MetS). Due to their body size, minipigs can provide abundant amounts of sample materials, available for extensive analyses by methodologically different approaches. DIO Göttingen minipigs display histomorphologically manifested multifocal adipose tissue inflammation and degeneration, exclusively occurring in the visceral (visc.) adipose tissue, but not in subcutaneous (s.c.) body fat depots [6]. In humans, visceral adiposity and adipose tissue inflammation are associated with many adverse metabolic effects, including insulin resistance [45], dyslipidemia [46], and hypertension [47]. They are also considered to play key roles in the pathogenesis of the (human) metabolic syndrome (MetS) [48], which is also significantly correlated with an increased risk of cardiovascular complications, such as myocardial infarction and stroke [49]. In obese patients with the MetS, as well as in murine DIO models, inflammatory adipose tissue alterations preferentially affect the visceral adipose tissue depot(s) [48]. The inflammation is (histo)morphologically characterized by increased numbers of lymphocytes and macrophages, along with locally and systemically increased levels of inflammatory cytokines, such as tumor necrosis factor alpha (TNFα) and interleukin 6 (IL-6), partially deriving from adipocytes [48,50]. The abundance levels of free fatty acids and of adipose tissue derived hormones, such as adiponectin and leptin are also altered in obesity and in the MetS [48,51,52]. Since many years, (translational) obesity research is focused on detection and characterization of obesity associated functional and molecular adipose tissue alterations, such as metabolic, lipidomic, transcriptomic and proteomic changes associated with the development of adipose tissue inflammation and the MetS. Different studies also examined morphological changes of adipocytes during development of adiposity, using different analysis approaches [12,53], such as flow cytometry of single isolated adipocytes and morphometric (incl. digital image) analysis of histological sections. These studies indicated a substantial role of adipocyte cell size in health, as well as under disease conditions [12]. In particular, visceral and subcutaneous adipocytes appear to react differentially towards adiposity, indicating a fat depot specific adipocyte growth reaction pattern [12,48], which could be relevant for the development of inflammatory alterations triggering the development of the MetS. However, obesity associated quantitative morphological parameters of adipocytes, such as “adipocyte size”, or differences between subcutaneous and visceral adipocytes, have yet not been extensively studied using unbiased quantitative morphological analysis methods.

In obesity-research, 2D-morphometric data obtained by planimetric analysis of adipocyte section profiles in histological sections of adipose tissue samples, such as the mean adipocyte section profile-areas/diameters, or the numbers of adipocyte section profiles per section area, are frequently used to describe adipocyte morphology [53]. These parameters can precisely and quickly be determined in standard histology sections, using digital image analysis software, such as “Image J”. Also, in the present study, the obesity-related changes of adipocyte morphology in s.c. and visc. adipose tissue depots of obese *vs*. lean minipigs were associated with significant increases of the mean adipocyte section profile areas and diameters, respectively, with significant decreases of the numbers of adipocyte section profiles per section area. However, 2D histomorphometric measurement data of cell section profiles are, offhand, neither equivalent to, nor can they be directly compared to 3D cell volumes and numbers, which represent inherently different parameters [8,39,54]. 2D histomorphometric measurements of cell section profiles in single histological sections can also not provide information about individual cell volumes, or the distribution of cell sizes in a defined tissue reference compartment. A precise, accurate, and objective characterization and differentiation of obesity-related hyperplastic and hypertrophic adipocyte growth patterns requires, by definition, unbiased measures of quantitative morphological parameters, namely the mean adipocyte volume, and the total number of adipocytes. However, these quantitative morphological parameters can generally not be determined without bias by 2D histomorphometric measurements of cell section profiles in single arbitrarily sampled histological sections [7–9,14,39,54]. Here, unbiased quantitative stereological methods, based on stochastic geometry, are required, representing the “gold-standard” for quantitative morphological analyses of biological tissues in biomedical sciences [7–9,14,33].

### Discussion of unbiased quantitative morphological analyses for determination of adipocyte volumes and numbers

The general principles and methodologies of quantitative stereological analyses and applicable sampling strategies have comprehensively been described in several textbooks [7,8,33,55], reviews and studies [9,14–17,28,39,42,54] the interested reader is referred to. Early “model-based” quantitative stereology methods use(d) (model-) assumptions about the shape, the shape- and size-distribution, the orientation, and the distribution of the examined tissue structures, to estimate their 3D volumes, numbers, surfaces, or lengths in histological sections. These model-based methods are therefore, independent how close the respective estimates may come to reality, inherently biased. In contrast, the accuracy and precision of estimates of stereological parameters obtained by modern, unbiased (design-based, model-free) quantitative stereological methods, such as the methods featured in the present work, do not depend on any model-assumptions, but only rely on adequate sampling designs and stereological probes. To obtain unbiased estimates of 3D morphological parameters of the tissue structures of interest (*e*.*g*., cell volumes, surfaces, lengths, and numbers), quantitative stereological analyses depend on “random sampling designs”, where samples are taken from strictly randomly selected locations (*i*.*e*., each location within a given tissue reference space has the same, random, chance (equiprobability) to be actually sampled) to appropriately generate representative samples that adequately reflect the interested features of the tissue reference compartment they are derived from [7,8,28]. Importantly, random-, or equiprobability-sampling designs do not require any external knowledge, such as size, shape, orientation, or spatial distribution of the objects of interest. The accuracy (error probability) of random sampling designs is predefined by the number and (fractional) size of the selected samples, relative to the size of the reference space, and to the abundance and distribution of the objects of interest within the reference space [42,56]. The efficiency of random sampling designs (*i*.*e*., the sampling effort, *i*.*e*., the necessary minimal number of samples) can significantly be improved, using systematic uniform random (SUR) sampling strategies [28]. In SUR-sampling designs, samples (of identical size) are selected from their corresponding reference space according to a random starting point but with a fixed, periodic (sampling) interval. SUR-sampling designs are appropriate to unbiasedly determine quantitative morphological features of biological structures. SUR-sampling designs are easy to apply, and, as long as the chosen sampling interval does not accidentally coincide with a cyclical pattern of the sampled structures, they eliminate the risk of preferential selection of objects with distinct properties, such as *e*.*g*., large volumes (*i*.*e*., a clustered selection, which would bias the obtained quantitative stereological estimates). In unbiased quantitative stereological analyses, SUR sampling designs are generally applied on all levels of sampling, *i*.*e*., to sample tissue-specimens, -blocks, and -sections, section fields of view, and test-fields/counting frames, warranting their unbiased representativity [8]. In contrast, “stratified sampling designs”, use prior knowledge for specification of particular properties/features to distinguish defined subpopulations of interested structures [42]. In stratified sampling designs, the sample size (and number) is adjusted in relation to *e*.*g*., the sizes, staining-intensities, neighborhood conditions, spatial distributions, or interspatial relationships of the objects of interest with distinct other tissue elements. Stratified sampling designs play an important role in image segmentation algorithms and for intensity threshold determination in immunohistochemically stained sections and are often applied in *e*.*g*., diagnostic (digital) pathology [42].

Unbiased quantitative stereological analyses estimate 3D morphological parameters (volumes, surfaces, lengths, and numbers of the tissue structures of interest) from their corresponding volume densities. These are determined, using specific test systems, stereological probes, and appropriately oriented 2D sections of the tissue, considering the effects of (potential) anisotropy, preferred orientation and non-uniform distribution of the tissue structure(s) of interest in their reference compartment [7,8]. Volume-, surface-, and length densities can be determined in single 2D tissue sections by appropriate stereological probes. In contrast, unbiased (number weighted) estimations of mean particle volumes and particle numbers require three-dimensional stereological test systems, such as the disector [7–9]. For calculation of absolute quantities, the volume-, surface-, length-, or numerical volume-densities are multiplied with the total volume of the corresponding reference compartment, which has to be determined in advance [16]. Some quantitative stereological approaches, such as determination of numerical volume densities with the physical disector [8,29], require consideration of the shrinkage of the tissue, associated with the embedding of tissue samples in histological embedding media [43]. Here, plastic embedding media, such as GMA/MMA, are preferable, since they allow preparation of thin sections and cause a precisely determinable, uniform, reproducible, and relatively low embedding related tissue shrinkage, as compared to paraffin embedding [7,35,43]. Regarding the orientation of the analyzed tissue sections, it needs to be noted, that determination of volume densities by point counting and analyses of numerical volume densities of particles with the disector can be performed in arbitrarily oriented sections (provided that unbiased, *i*.*e*., representative, randomly sampled sections are analyzed, and that the examined structures of interest and their tissue reference compartment are not affected by differential embedding related shrinkage), whereas other quantitative stereological analysis parameters, such as surface- and length-densities may require a randomization of the section plane orientation [8]. For estimation of surface densities, for example, vertical sampling designs are commonly applied [57], whereas estimates of length densities require sections with random orientation in all three dimensions of space (isotropic uniform random, or IUR sections) [8,32,40,58]. Although determination of V_V(AC/ATD)_ and N_V(AC/ATD)_ do not require IUR sections, it might though be useful to apply IUR sampling designs, since all quantitative stereological parameters can be analyzed in IUR sections, thus allowing for a retrospective analysis of any additional stereological parameters in the same sections, without the necessity to generate new sections with randomized section plane orientations.

The analysis of individual particle volumes, such as individual cell volumes within the tissue, still remains a challenging and extremely laborious task, using unbiased quantitative stereological analysis methods [8]. Indeed, the determination of individual cell volumes (unbiased by the volume of the analyzed cell) within a tissue sample, requires the analysis of the cells section profile areas in approximately equidistant, parallel, serial sections, completely covering the entire vertical extension of the cell (Cavalieri’s principle) [8]. Since unilocular adipocytes in white adipose tissue are comparably large cells (with an average diameter of often > 150 μm), this procedure requires the analysis of a large number of serial sections, and is most cumbersome, since identification of corresponding section profiles of the same cell in different sections becomes increasingly difficult, as higher the vertical distance of the two examined sections is. Therefore, individual fat cell volumes determined by unbiased quantitative stereological methods have yet not been reported (whereas, some studies determined individual volumes of adipocytes in single cell suspensions generated by enzymatic separation of adipocytes from adipose tissue samples [12,59]).

In the present study, strict sampling designs were applied for quantitative stereological analyses of adipocyte sizes, numbers, and mean volumes, to characterize the principal obesity associated quantitative morphological alterations of adipocytes in different adipose tissue depots of the examined DIO minipig model. The volume densities of adipocytes in the different adipose tissue depots were determined by point counting in arbitrarily oriented paraffin sections of SUR sampled adipose tissue specimens. In tissues where differential shrinkage is not relevant, this parameter is independent of the extent of embedding related tissue shrinkage and can be determined without greater effort, using standard paraffin sections (volume density determination is of course also possible in sections of plastic embedded tissue samples, or in (virtual) 2D optical LSFM sections, if paraffin sections are not prepared in a given study). For determination of individual adipocyte volumes and the numerical volume densities of adipocytes within their corresponding adipose tissue depots by LSFM-based morphological analysis, the disector method [8,29,35] was chosen, because it warrants an unbiased, number weighted, and swift sampling of adipocytes for subsequent analyses directly in the volume rendered 3D reconstruction of the LSFM imaged adipose tissue sample. For the confirmative unbiased quantitative stereological analysis approach, the same stereological probe was used, although the physical disector method is often considered technically complex and time consuming, as compared to alternative unbiased quantitative stereological methods, such as *e*.*g*., fractionator sampling designs combined with the optical disector and the nucleator [7,8]. In the present study, the physical disector method was, subjectively, still found absolutely suitable for the unbiased quantitative stereological analysis of adipose tissue specimen sampled from large body fat depots. Next to the application of appropriate sampling procedures, there are essentially two relevant, technical factors that must be adequately determined to warrant the accuracy of particle volume- and number estimates obtained by physical disector analyses: the extent of embedding-related tissue shrinkage and the factual thicknesses of the examined sections [30,35]. In the present study, the (soft) adipose tissue samples were shape-stabilized by encasing in agar to prevent mechanical deformation/compression during the embedding process [30]. Embedding-related tissue shrinkage and section-thicknesses were precisely determined using established volumetric measurement methods [30,31], and reflectometric measurement analysis [35], respectively, and correspondingly used for calculation of the numerical volume estimates of adipocytes in their adipose tissue reference compartments. The homogeneity of the extent of 3D-shrinkage related to GMA-MMA-embedding of adipose tissue samples was confirmed by the low standard deviation of shrinkage rates of individual samples.

### Discussion of LSFM-based unbiased quantitative morphological analyses of adipocytes

Although the featured unbiased quantitative stereological analysis methods allow for an accurate estimation of relevant morphological adipocyte parameters, the application of these methods remains time intense and cumbersome. It might be for this reason, that unbiased quantitative stereological analyses have only rarely been implemented to characterize adipocyte morphology in previous studies examining human specimens [60], or translational obesity models [61]. Therefore, we tested the applicability of 3D light sheet fluorescence microscopy (LSFM) for quantitative morphological analyses of optically cleared adipose tissue samples, as an efficient alternative to 2D section based unbiased quantitative stereological analysis approaches. To evaluate the comparability of the results of LSFM-based quantitative morphological analyses of cleared adipose tissue samples to the results obtained by unbiased stereological methods, both analysis approaches were performed on adipose tissue specimens SUR sampled from the same adipose tissue depots of the identical pigs. Previously, few other studies have already successfully applied LSFM analyses for examination of distinct quantitative morphological parameters in other types of tissues, *e*.*g*., to count the number of glomeruli in murine kidneys [62], to analyse the volume of bronchus associated lymphoid tissue in mice [63], or to determine the volume and surface of fish gill lamellae [64]. A large variety of different techniques for tissue clearing has been developed [65], such as CLARITY, iDISCO, 3DISCO, FRUIT, CLEAR T [20], vDISCO [66], Adipo Clear [27], and ECi [62], optimized for different sample types and various downstream applications. For quantitative morphological analyses of adipose tissue samples, we used the 3DISCO technique [25], since this clearing method works fast and produces firm, dimensionally stable, but easy to cut specimens up to the size of several cubic centimetres. 3DISCO clearing is generally known to cause a considerable shrinkage of the cleared tissue samples [67]. In the present study, however, a constant and regular volume decrease of adipose tissue samples of approximately ~21% was associated with the applied clearing and staining procedure, as determined by precise volumetric measurement methods. Interestingly, this degree of clearing related tissue shrinkage is approximately the same, as the extent of embedding related tissue shrinkage of GMA/MMA embedded adipose tissue samples (which is generally considered to be beneficially low, as compared to other histological embedding media, such as paraffin). Other solvent based clearing protocols, such as iDISCO+ or Adipo-Clear reportedly cause a generally lower degree of clearing associated tissue shrinkage [27,67,68] than the 3DISCO procedure and have been successfully applied for clearing and 3D LSFM imaging of adipose tissue samples [27]. For quantitative morphological analyses of adipose tissue samples, these clearing protocols might thus represent suitable alternatives to the 3DISCO method.

In 3DISCO cleared adipose tissue samples, the (adipocyte) cell membranes displayed a variably strong autofluorescence at 520/40 nm (excitation range) / 585/40 nm (emission range) wavelength. The automatic digital image analysis of single adipocyte cell volumes in 3D reconstructions of cleared adipose tissue samples depends on the separation of cell membrane staining signals and the unstained inner space of the cell [69]. The adipocyte cell volume is calculated from the number of voxels within the unstained (*i*.*e*., optically empty background) centre of the cell that is completely surrounded by a continuously stained cell membrane. In unstained 3DISCO cleared adipose tissue samples, the cell membranes of single adipocytes could often not be detected in complete continuity, thus hindering single cell volume analyses (if the cell membrane staining between two adjacent adipocytes is disrupted, these two cells will be detected as one, thus producing a false individual cell volume measurement value). To enhance the adipocyte cell membrane autofluorescence in the 3DISCO cleared tissue samples, a simple eosin (post) staining was applied. In 3DISCO cleared and eosin-stained samples all adipocyte cell membranes were reliably and sufficiently strong labelled. The samples could straightforwardly be imaged by LSFM at 585/40 nm wavelength (emission range), with a 0.63–10 magnification and a z-step height (*i*.*e*., vertical resolution) of 5 μm. A standard LSFM image viewing and analysis software (arivis Vision4D vers. 3.0) was used to generate precise 3D sample reconstructions [69]. After digital adjustment of the staining intensity to limit the width of the stained adipocyte cell membranes to < 1 μm, all individual adipocytes present in the examined tissue sample could be unambiguously identified.

In 3D fluorescence microscopy, the accuracy of quantitative measurements of object volumes in 3D digital images reconstructed from z-stacks of 2D optical (fluorescence) images depends on the accuracy of the 3D image reconstruction, *i*.*e*., a precise match of the volumes (and shapes) of the imaged objects and their digital reconstructions. Major factors causing distortion of object shapes (and correspondingly of their measured volumes) in 3D reconstructions of 2D fluorescent image piles (z-stacks) are a nonconformity of the nominal and factual z-step size (*i*.*e*., an untrue vertical scaling), and imaging artifacts due to spherical aberration (SA) due to refractive index (RI) differences between the imaged sample and the surrounding medium (clearing/mounting medium, air, objective lens), as well as between different lens elements inside a fluorescence microscope [44]. Light rays passing such RI-mismatched interfaces are focused to different points of the optical axis, causing a shift of the optical focus plane of the imaged object. During z-stack acquisition of fluorescent images, the vertical distance that the imaged object physically moves (*i*.*e*., the stage movement) in every z-step, is then different from the movement of the acquired image (focus) planes, leading to artificially distorted (elongated or compressed) 3D object images, and correspondingly to incorrect object volumes [44]. In 3D LSF-microscopy, shape and volume distortions of imaged objects can be generally addressed by using appropriate technical equipment (such as SA-corrected LSFM-objectives) and precisely adjusted instrument settings (precise calibration of z-step height, alignment of light sheet planes, adjustment of light sheet widths and focus settings, *etc*.). SA-imaging artifacts due to RI-mismatches can be avoided by using dipping lenses (or dipping caps) and immersion media with RIs adapted to the RIs of the cleared tissue samples, and by correction of SA-induced digital image distortion with appropriate algorithms [44]. In a given experiment, a potential distortion of images of biological objects of interest (such as adipocytes) can also be examined by imaging of control (test)-objects with similar properties (shape, size, RI, fluorescence), using the same instrument settings for image acquisition, volume rendering and image analysis. In the present study, diverse technical and methodological precautions were taken to avoid image-distortion and correspondingly incorrect adipocyte volume measurement results, including precise control of the z-step advance, adequate adjustment and control of LSFM-instrument settings and image (pixel/voxel-size) scaling. To minimize SA-imaging artifacts due to RI-mismatches, the objective was equipped with an optimized dipping cap, and the same chemical (BABB, RI: 1.56) [70] was used as solvent for clearing, and as submersion medium for imaging of adipose tissue samples. The realistic rendering of object shapes and volumes in digital 3D-LSFM reconstructions was additionally confirmed by imaging and volumetric image-analysis of fluorescently labelled, spherical, polystyrene (RI: 1.59) microparticles (*i*.*e*., comparable to the RI of solvent-cleared tissue samples [44]) with precisely defined diameters of ~100 μm (*i*.*e*., comparable to the size of adipocytes), using the exactly same instrument-, image-acquisition, and analysis-settings, as for analysis of the adipose tissue samples.

For determination of V_V(AC/ATD)_ and N_V(AC/ATD)_ in 3D LSFM autofluorescence images of the optically cleared adipose tissue samples, the same stereological probe (*i*.*e*., disector analyses in SUR sampled fields of view) was applied, as in the unbiased quantitative stereological analysis approach, ensuring the unbiasedness of the quantitative morphological LSFM measurements.

The used arivis Vision4D (vers. 3.0) imaging and analysis software does not feature integrated specific application tools for SUR sampling, point counting, disector analyses with unbiased counting frames, or other quantitative stereological probes [69] (*therefore*, *in the view of the authors*, *a “stereology-add-on” for the arivis Vision4D software would be beneficial*). However, the application of these stereological probes was supported by advantageous functions of the arivis Vision4D software, such as the “split screen” function, or the “*Magic Wand*” tool. The split screen function allowed for a side-by-side view of two analysis windows of the same 3D LSFM dataset, enabling a parallel, independent view of two different, determinable, optical section planes of the identical 3D tissue sample reconstruction for the disector. Combined with the “*Magic Wand*” tool, for labelling of individual cells in the 3D sample reconstruction, an unbiased, number weighed disector sampling of adipocytes could efficiently be performed. The “*Magic Wand*” tool also allowed a rapid analysis of the individual cell volumes (and other quantitative morphometric parameters, such as surface area) of the sampled adipocytes.

The (shrinkage corrected) measurement values of adipocyte numbers and volumes in s.c.- and visc.-adipose tissue samples of lean and obese pigs assessed by LSFM-based analyses and by unbiased quantitative stereological analyses were virtually equal, thus proving the suitability of LSFM-based analyses for precise and unbiased determination of relevant quantitative morphological adipocyte parameters.

### Discussion of the advantages of LSFM-based analyses of adipocyte volumes and numbers

In contrast to the elaborate tissue processing and analysis steps associated with 2D section based unbiased quantitative stereological analyses (plastic-embedding of tissue samples, preparation of thin section series, analysis of section thicknesses, physical disector analyses), the clearing, LSFM imaging and subsequent digital quantitative morphological analyses of adipose tissue samples required significantly less time, workload, and material costs. Particularly, the straightforward disector-sampling process of adipocytes in virtual 3D-adipose tissue reconstructions and the direct digital volume analysis of the sampled adipocytes was (subjectively) far less cumbersome and more certain than the sampling and counting process of adipocyte nuclei in physical disector section pairs. Most remarkably, LSFM analysis also enables unbiased measurements of individual adipocyte volumes. In contrast, determination of individual fat cell volumes with (2D-section-based) quantitative stereological analysis methods has so far not been possible with reasonable technical effort. Due to the (comparably large) size of adipocytes, the applied vertical resolution (step size) of ~5 μm was small enough to provide sufficiently accurate measurements of individual adipocyte cell volumes in 3D LSFM image reconstructions. Thus, for the first time, it is now possible to monitor not only obesity associated changes of the mean adipocyte cell volume within intact tissue samples, but also to detect changes in the size distribution of the adipocytes in a defined adipose tissue depot (which even might not cause a net change of the mean fat cell volume). This way, subpopulations of adipocytes could be identified, which might display differential hyperplastic and hypertrophic growth reaction patterns, metabolic capacities and pro-inflammatory properties in different body fat depots in states of hypercaloric nutrition and developing obesity [12].

### Discussion of obesity-associated, fat depot specific alterations of adipocyte growth patterns in the Göttingen minipig DIO model

The results of the present study objectively prove, that the obesity related volume increase of the subcutaneous and visceral adipose tissue depots in DIO Göttingen minipigs is due to different, depot specific adipocyte growth reaction patterns. Adipocytes in the subcutaneous adipose tissue of obese animals displayed a comparatively balanced ratio of hyperplastic (*i*.*e*., an increase of cell numbers) and hypertrophic (*i*.*e*., an increase of the cell size, characterized by the mean adipocyte volume) growth reaction patterns. In contrast, the adiposity related increase of the visceral adipose tissue depot in obese animals was characterized by a disproportionally marked increase of the mean adipocyte volume, paralleled by an only minimal and insignificant increase of the total number of adipocytes. In the present study, the largest adipocytes in the visceral adipose tissue depot of obese pigs displayed individual cell volumes of more than 15 x 10^6^ μm^3^, exceeding the mean volume of subcutaneous adipocytes in obese minipigs by more than 7-fold. The morphologically different growth reaction patterns of subcutaneous and visceral adipocytes during development of obesity might provide a possible explanation for the pathogenesis of the inflammatory lesions exclusively occurring in the visceral adipose tissue. An excessive (individual or general) enlargement of adipocytes might disturb the (mechanical) stability of the adipose tissue and expose enlarged adipocytes to increased shear stress [12,71,72], as soon as a “critical” cell size is reached. This might probably induce altered adipocytokine expression patterns, triggering the development of an inflammatory milieu with activation of resident macrophages and attraction of infiltrating inflammatory cells, or directly lead to adipocyte degradation [12]. Previous studies have already demonstrated a correlation between obesity associated adipocyte cell hypertrophy and impaired insulin mediated glucose uptake and increased lipolysis [73], predicting pathological conditions such as insulin resistance and type 2 diabetes [73–77]. Obesity related adipocyte hypertrophy was also shown to be positively correlated with the expression of inflammatory genes [78], increased pro-inflammatory adipokine secretion (*e*.*g*., IL-6, IL-8, MCP-1) [79], and reduced secretion of anti-inflammatory adipokines (*e*.*g*., adiponectin), contributing to an increased inflammatory response in adipose tissue [80].

Previous studies have indicated that not only the metabolic state and the pro-inflammatory attributes of hypertrophied adipocytes are altered in states of obesity, but also those of increased proportions of small adipocytes with impaired adipogenesis and/or terminal differentiation [81–83]. In the examined DIO minipig model, the relative size distribution patterns of individual adipocytes in s.c.- and visc.- adipose tissue depots did not considerably vary between lean and obese pigs. These findings indicate a largely homogenous volume increase of individual visceral adipocytes during development of adiposity, exclusively mediated by cellular hypertrophy. In contrast, the normal distribution of individual adipocyte volumes in the subcutaneous adipose tissue of obese minipigs was maintained by balanced hypertrophic and hyperplastic adipocyte growth patterns. Thereby, these findings also demonstrate that significant changes of either cell numbers or cell volumes do not necessarily also manifest in altered size distributions, or *vice versa*.

### Limitations of the study

Currently, the technical equipment, the configuration of hardware elements, and the performance capabilities of different LSFM-platforms in different laboratories are not standardized. Different optical parameters of the installed objectives (dry-lenses *vs*. dipping-lenses, different magnification-ranges and numerical apertures, adaption of objectives to immersion liquids of distinct refractive index-ranges, *etc*.), variable light sheet dimensions and geometries, different emission/excitation wavelength ranges, different instrument settings (including z-stage calibration), and camera properties might cause variable image qualities, resolution capacities, and potentially imaging artifacts. These factors can not only impede accurate quantitative measurements of adipocyte volumes, but also hinder a precise reproduction of LSFM-based quantitative morphological measurement results with different LSFM-systems. In a given study, LSFM-based quantitative morphological analyses of different adipose tissue samples should therefore be performed on identically processed tissue samples, using the same LSFM-instrument and analysis settings. Since a standardization of the currently used LSFM-systems and the optical properties of their components is yet not achieved, any studies reporting quantitative morphological measurement data obtained by LSFM-analysis should precisely indicate all relevant technical details of the used LSFM-instrument. Additionally, the experimental design of such studies should schedule LSFM-imaging and analysis of appropriate shape- and volume controls (*i*.*e*., test objects, such as the fluorescent polystyrene beads used in the present study) to confirm the general correctness of 3D-LSFM measurement results.

As a second seeming limitation of the described LSFM-approach, adipocytes are only identified by their cell-membrane autofluorescence signal (intensified by eosin-staining). Differentiation of adipocytes and other tissue structures, such as vessels, strands of connective tissue (**Fig 4B**), or other cell types, thus solely depends on their characteristic shape. However, this theoretical disadvantage is compensated by the possibility to watch the tissue/cell morphology in 3D. For the purpose of volume analysis of (unilocular) adipocytes in cleared adipose tissue samples, detection of cell-membrane autofluorescence by LSFM (without additional elaborate and expensive cell-type specific labelling procedures) was therefore considered a fast, efficient, and absolutely sufficient approach. However, if cell nuclei or different cell types/tissue structures have to be specifically labeled and analyzed in an LSFM-experiment (*e*.*g*., for differentiation of different subtypes of uni-or multilocular adipocytes, or inflammatory cells), other appropriate clearing methods and labelling protocols have to be applied and adapted to the given experimental design [27,41].

Finally, the present study examined adipocyte morphology in a pig-model of DIO, whereas the majority of translational obesity research is currently performed using rodent models. The application of the described LSFM-approach for adipocyte volume- and number analysis in *e*.*g*., mice would therefore require an adaption of the used (SUR)-sampling regime to the smaller volume of the examined adipose tissue depots and the corresponding numbers of sampled tissue locations. However, application of the described clearing/staining protocol and the LSFM-imaging- and analysis-procedures is principally also possible in murine adipose tissue samples, as murine and porcine unilocular adipocytes display a similar morphology (**S5 Fig**) and their cell volumes are principally in the same order of magnitude.

### Perspectives of LSFM-based quantitative morphological adipocyte analyses

Next to the determination of adipocyte volumes and numbers in cleared adipose tissue samples, 3D-LSFM analyses also provide the possibility for comprehensive analyses of additional qualitative-, and quantitative morphological adipocyte parameters, such as shape, shape variability, surface, isotropy, *etc*. Alterations of these parameters might also be relevant for adipocyte (dys)function in obesity (*e*.*g*., by affecting the shear-stress-stability of hypertrophied adipocytes), and/or used as morphological markers of metabolic disease states in translational obesity models. In future studies, combination the described LSFM-approach for adipocyte volume- and number analyses with additional specific fluorescence labelling and tissue clearing techniques will also allow detailed quantitative and spatial analyses of different cell populations in adipose tissue samples, *e*.*g*., by using specific markers of white (unilocular), beige, or brown (multilocular) adipocytes, or different inflammatory cell types.

## Conclusion

LSFM-based analyses can be recommended as an elegant and efficient approach for comprehensive quantitative characterization of adipocyte morphology in future studies, probably also in other species. Of note, LSFM of 3DISCO cleared tissue samples can also be combined with other imaging modalities, such as histology, immunohistochemistry, or mass spectrometry imaging, and also works using paraffin-embedded tissue samples, *e*.*g*., from pathology archives [84]. The swift determinability of accurate numerical values of adipocyte numbers and (individual) volumes can undoubtedly significantly contribute to increase the effectiveness of a study, *e*.*g*., by correlation of quantitative morphological adipocyte parameters with transcript profiling-, proteomic-, lipidomic-, or metabolomic analysis data, or measurements of inflammatory cytokines and hormones analyzed in the same adipose tissue samples, or with systemic marker concentrations, or clinical outcomes. We are currently considering performing such multi-OMICS studies on biobank adipose tissue samples of our DIO minipig model to further clarify the relationship(s) between obesity associated morphological changes of fat cells and their corresponding molecular function profiles in defined adipose tissue depots.

## Supporting information

S1 Fig Systematic uniform random (SUR) sampling for generation of representative adipose tissue samples for downstream quantitative morphological analyses. (TIF)

S2 Fig Determination of the volume density and total volume of adipocytes in adipose tissue depots. (TIF)

S3 Fig Estimation and correction of embedding related tissue shrinkage. (TIF)

S4 Fig Control of correct LSFM-image (pixel/voxel-size) scaling/calibration for realistic object shape- and volume-rendering in digital 3D-LSFM image reconstructions. (TIF)

S5 Fig Demonstration of the principal similarity of the morphology of adipocytes in histological sections and LSFM-images from murine and porcine subcutaneous adipose tissue samples. (TIF)

S1 Video LSFM 3D reconstruction of a 3DISCO cleared porcine subcutaneous adipose tissue sample. (MP4)

S1 File Step-by-step protocol for disector sampling and volume analysis of adipocytes with the arivis Vision4D imaging and analysis software. (DOCX)

S2 File Exemplary LSFM-dataset. Downloadable from: doi:10.5061/dryad.8gtht76nt.
(DOCX)
