# Supplementary figures and images for: Unbiased analysis of obesity related, fat depot specific changes of adipocyte volumes and numbers using light sheet fluorescence microscopy
Source: PLoS One. 2021 Mar 16;16(3):e0248594. doi: 10.1371/journal.pone.0248594 (PMC7963095; doi:10.1371/journal.pone.0248594)

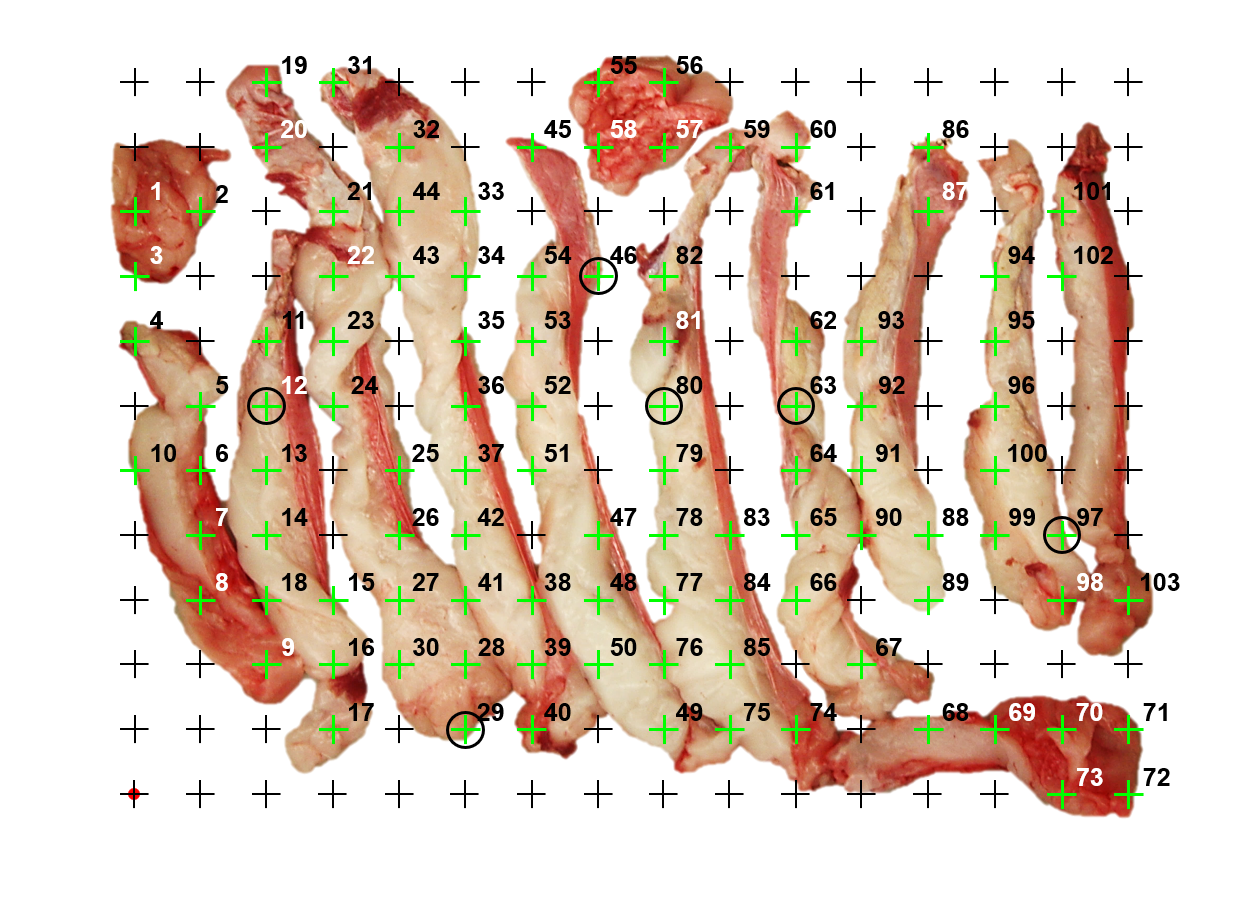

Supplement: S1 Fig — (TIF) [file pone.0248594.s001.tif]

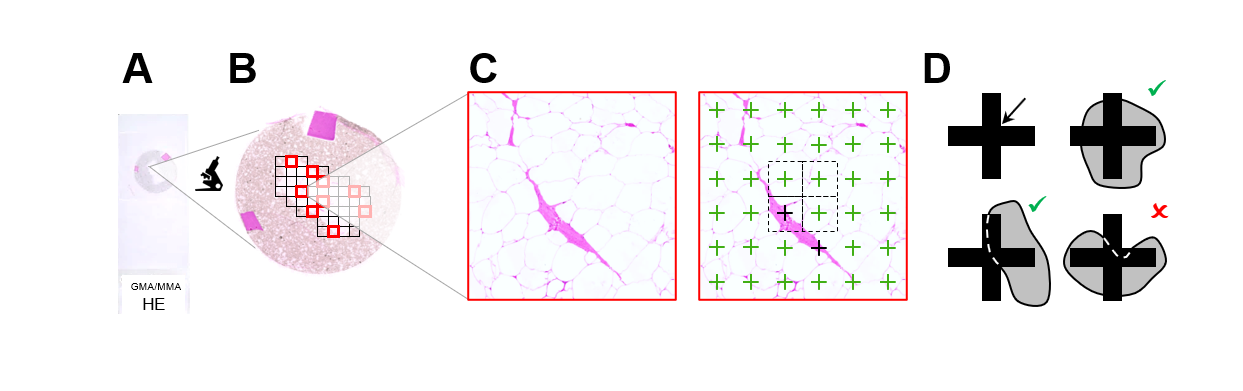

Supplement: S2 Fig — (TIF) [file pone.0248594.s002.tif]

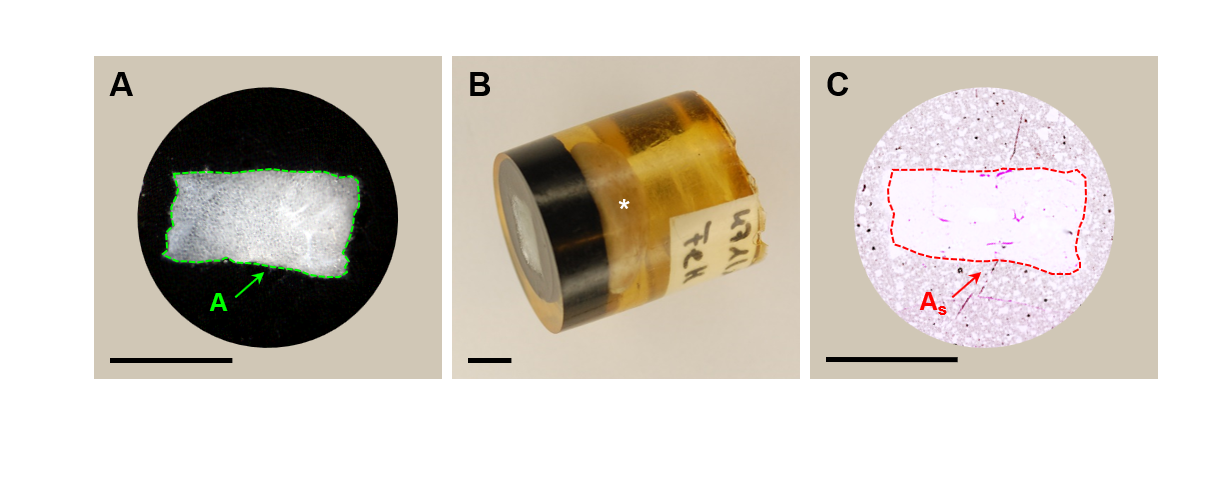

Supplement: S3 Fig — (TIF) [file pone.0248594.s003.tif]

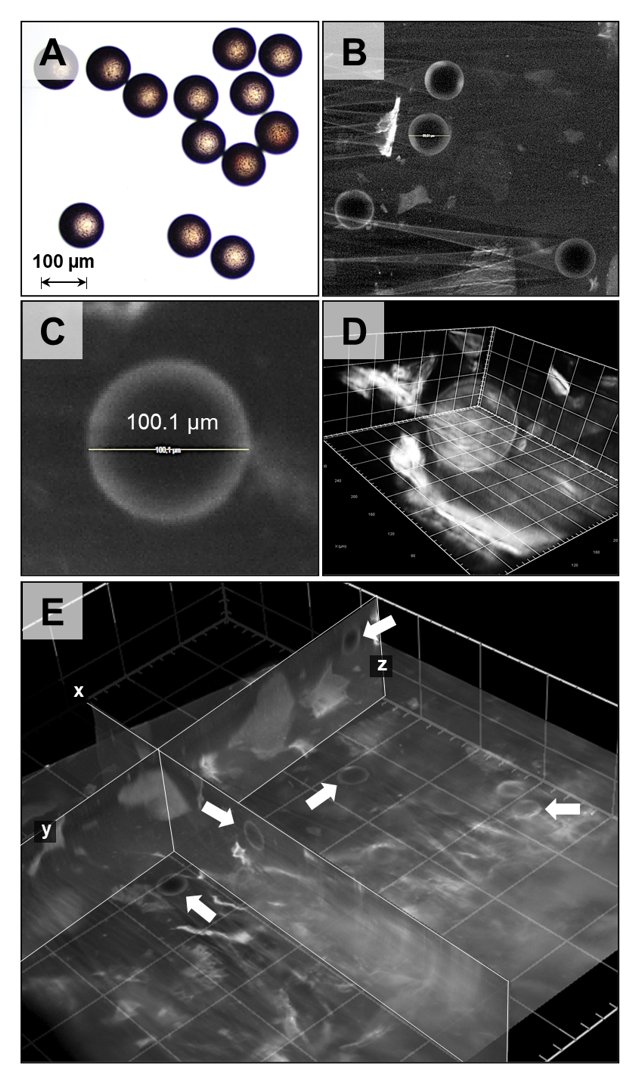

Supplement: S4 Fig — (TIF) [file pone.0248594.s004.tif]

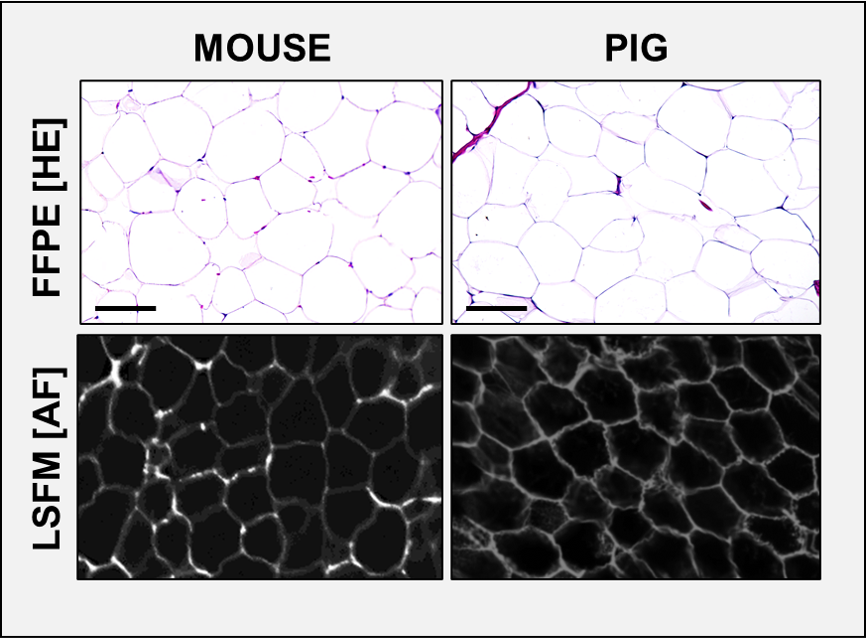

Supplement: S5 Fig — (TIF) [file pone.0248594.s005.tif]
