# Supplementary material for: Unbiased analysis of obesity related, fat depot specific changes of adipocyte volumes and numbers using light sheet fluorescence microscopy
Source: PLoS One. 2021 Mar 16;16(3):e0248594. doi: 10.1371/journal.pone.0248594 (PMC7963095; doi:10.1371/journal.pone.0248594)
Supplement: S1 File — (DOCX) [file pone.0248594.s007.docx]

**Step-by-step protocol for unbiased sampling**

**and volume analysis of adipocytes in 3D LSFM image reconstructions,**

**using the arivis Vision4D imaging and analysis software**

This protocol describes the generation of 3D LSFM image reconstructions from z-stacks of 2D LSFM-images of cleared adipose tissue samples using the arivis Vision4D (arivis, Germany) imaging and analysis software (I), as well as unbiased sampling of adipocytes with the disector method (II), and the direct 3D digital image analysis of individual adipocyte volumes with the “*Magic Wand*” tool (III). For practical exercise, a training dataset (Supporting Information S2 file) from the present study [containing 181 2D-LSFM-z-stack-images acquired in a 3DISCO-cleared, eosin-stained, s.c. adipose tissue sample of an obese Göttingen minipig at Ex/Em: 520/40nm/585/40nm with a z-step height of 5 µm] can be downloaded from: doi:10.5061/dryad.8gtht76nt.

*[The present protocol is only intended to serve as a supporting information protocol describing the application of methods featured in the corresponding publication by Theobalt et al. (2021). The protocol is neither an officially authorized publication of the arivis AG, nor does any of the authors have any commercial association with this company. For more detailed information on the applicability of the software, computer-system requirements, or available trial versions, the interested reader is referred to the arivis Vision4D homepage (*[*https://www.arivis.com/en/*](https://www.arivis.com/en/)*), and the arivis Vision4D operation manual and video-tutorials (*[*https://imaging.arivis.com/en/imaging-science/arivis-vision4d-video-tutorials*](https://imaging.arivis.com/en/imaging-science/arivis-vision4d-video-tutorials)*).]*

**I. Generation of 3D LSFM image reconstructions of cleared adipose tissue samples**

1. Open the arivis Vision4D imaging and analysis software (version 3.0 or higher).
2. Drag-drop the folder with the 2D LSFM z-stack images to the opened arivis Vision4D window. An “*Import files – Assume same structure for all files?*”-window will pop up. Klick “*Yes*”. Then a “*Import files –Select import destination*”-window will open. Choose “*New File*”, select folder and file name and klick “*OK*” (During import of the training dataset, an “*Import files –Select import scenario and destination*”-window will open. Choose the *“images as planes”* scenario as well as “*New File*”, select folder and file name and klick “*OK*”). A pop-up window will appear, showing the progress of image import. The software will create an SIS file (as well as a corresponding “*metadata*” and “*objects*” file) from each imported z-stack. By double-clicking on the SIS file, the imported dataset can be re-opened in the last saved setting (if the corresponding “*metadata*” and “*objects*” files are present in the same folder) without having to import the z-stack again.
3. After the image z-stack is loaded, a 2D view of the median image plane will appear (**Screenshot 1A**). Use the “*Zoom to Fit*” button (encircled in red) to display the entire image on the screen. Image properties (gamma-correction, contrast. brightness, color-channels, *etc.*) can be changed using the controllers/sliders displayed in the windows on the right side of the screen. The image plane displayed in the left screen window is set using the slider (red arrow) in the “*Navigator*” window (black arrow). In the presented example, fluorescence signals (of adipocyte membranes) are displayed in the grey-color channel (one channel) in the AUTO-intensity mode (red arrow). By default, the medial image plane of the z-stack (here: N° 147) is displayed.

Usually, the correct pixel size-scaling is automatically directly acquired from the metadata of the imported LSFM-image z-stack file. Using the training dataset S2 file, the correct pixel-scaling might, however, be reset to the default value of 1 µm in each direction of space. Therefore, the correct pixel size scaling for this dataset must be controlled in advance (**Screenshot 1B**). This is done by clicking on the *“Data”* button and then selecting *“Pixel Size”* in the pull-down menu (red arows). Enter the correct pixel sizes for the training dataset S2 file (X: 755,112 nm, Y: 755,112 nm and Z: 5) and klick on “*Change pixel size*” (arrow).


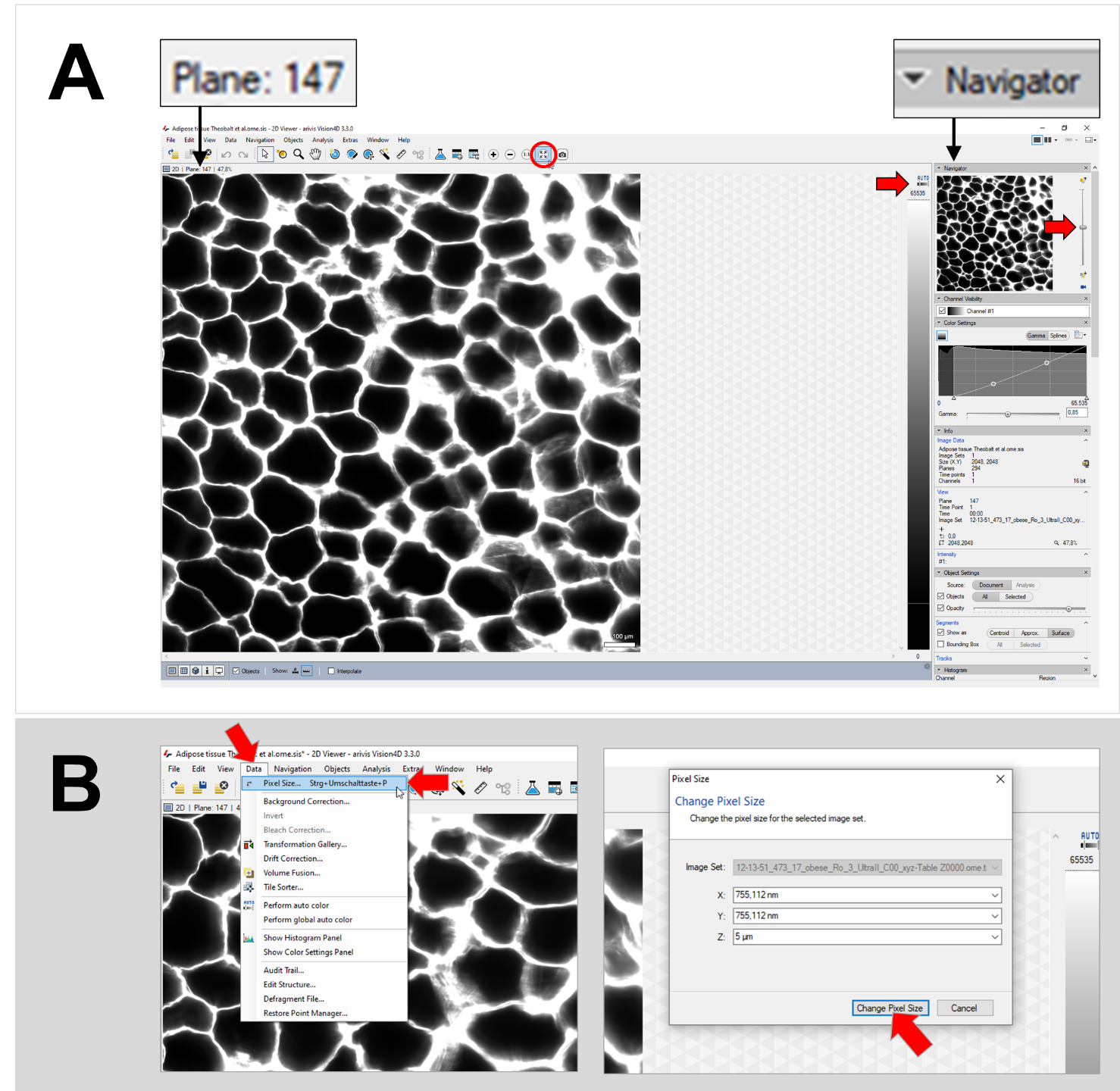


**Screenshot 1.**

_________________________________________________________________________________

**II. Unbiased sampling of adipocytes in 3D LSFM images with the disector method**

*For measurement of individual cell volumes, adipocytes are unbiasedly sampled in the 3D LSFM image of the adipose tissue sample, using the disector method. This sampling design warrants that the selection of adipocytes is not biased by their volume (size), shape, or orientation (i.e., that the adipocyte volume measurement results are not biased). The principle of the disector method is extensively explained in the main paper.*


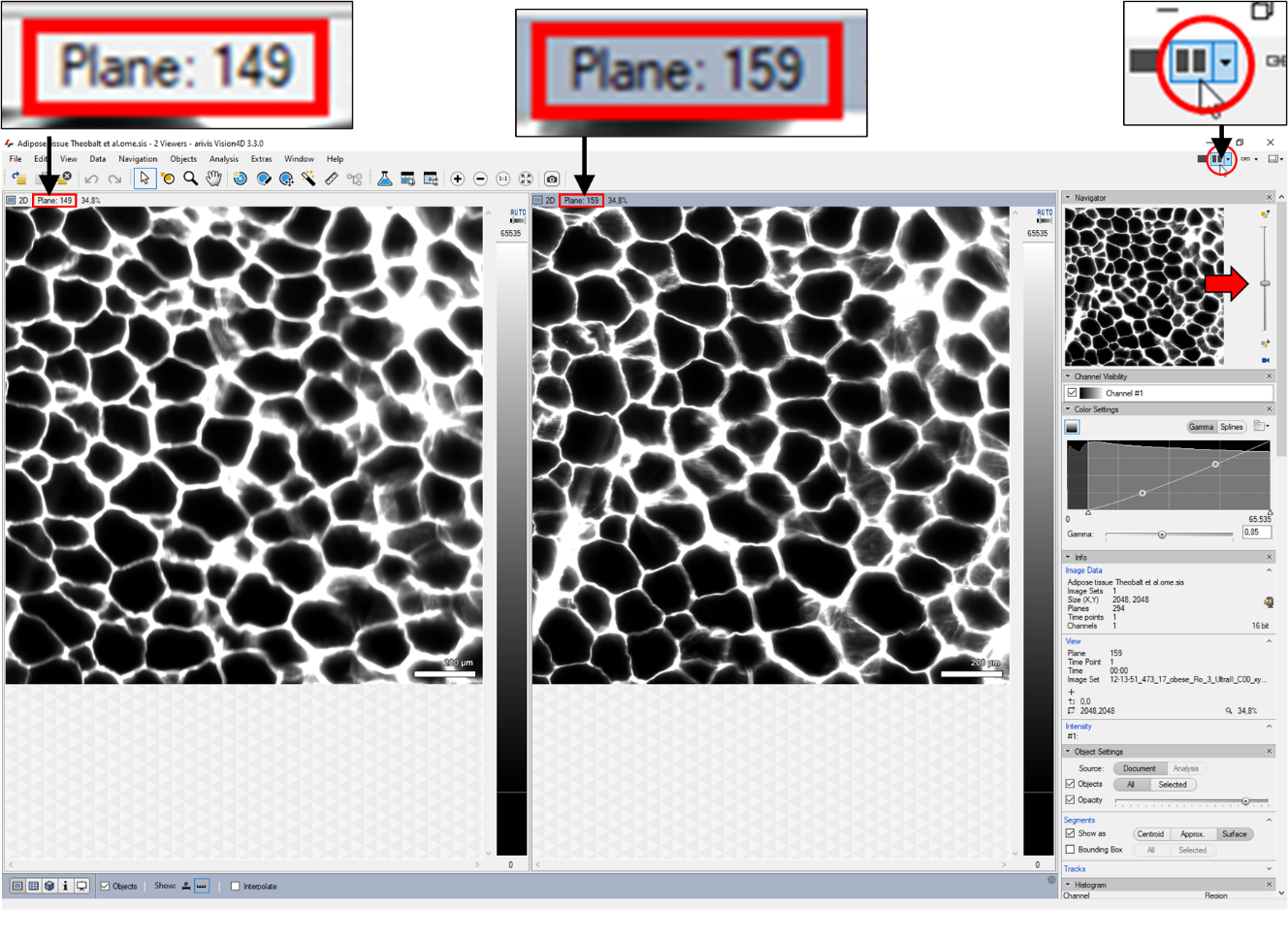


**Screenshot 2.**

1. Select the split-screen modus (encircled in red) for parallel display of two different section planes (**Screenshot 2**). To display both images in the same size, select each window and press the “*Zoom to Fit*” button (see I.3, **Screenshot 1A**).
2. Select a random section plane as the disector reference section plane (random sampling can be performed, using a random number table from an internet-random number generator). This section plane should be sampled from the middle of the z-stack (*i.e.,* in a range starting from approximately 40 section planes after the first, and 40 section planes before the last section plane of the image z-stack) to warrant that all section planes of the sampled adipocytes are completely present within the image set. Here, section plane N° 159 (black arrow) is sampled as the reference section plane (right screen window, the darker grey-blue color of the window frame indicates that this window is currently active).
3. Then switch to the left screen window (*i.e.,* the second section plane window) and systematically determine the disector look-up section plane considering the applicable disector height (*i.e.,* the distance between the reference- and the disector look-up section plane). The disector height should be approximately 1/3^rd^ of the minimal linear orthogonal projection of the measured objects (*i.e.,* 1/3^rd^ of the minimal adipocyte diameter – here, a disector height of 50 µm was chosen, referring to 10 section planes with an average single section thickness (*i.e.,* z-step height) of 5 µm), and section plane N° 149 was sampled as disector look-up section plane (black arrow). Use the slider in the “*Navigator*” window (red arrow) to move to the determined disector look-up section image plane.


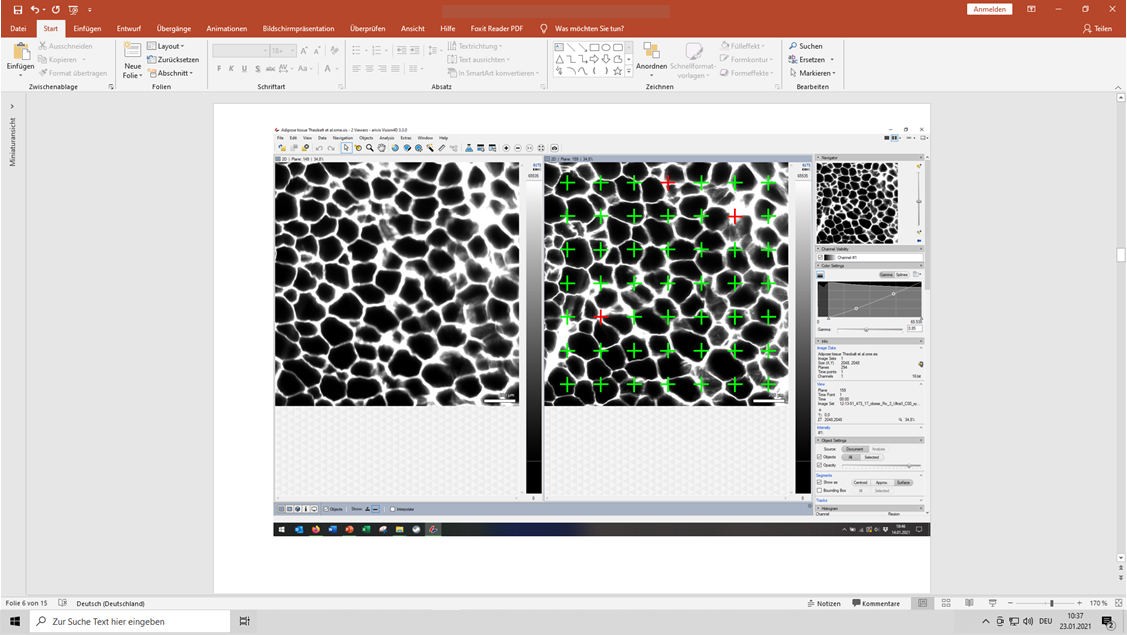


**Screenshot 3.**

1. Optionally (if not performed in paraffin sections) the area density of adipocytes in the adipose tissue can be determined by point-counting for estimation of the volume density of adipocytes in the adipose tissue. Since the arivis Vision4D imaging and analysis software does not provide a tool for superimposing cross grids, a screenshot of the image of the disector-look-up section is copied into another software-application (*e.g.,* Microsoft PowerPoint) and superimposed with a grid of equally spaced test points/crosses (**Screenshot 3**). The number of crosses hitting adipocyte section profiles (green) are counted (points are counted, if the upper right corner of a cross hits the structure of interest, *i.e.,* an adipocyte cross section profile) and this number is related to the total number of crosses hitting the adipose tissue (here, crosses hitting non-adipocyte cross sections within the adipose tissue, such as connective tissue strands, are displayed in red color). Here, 46 crosses of a 7x7 (49) point cross grid hit adipocyte cross section profiles.

**
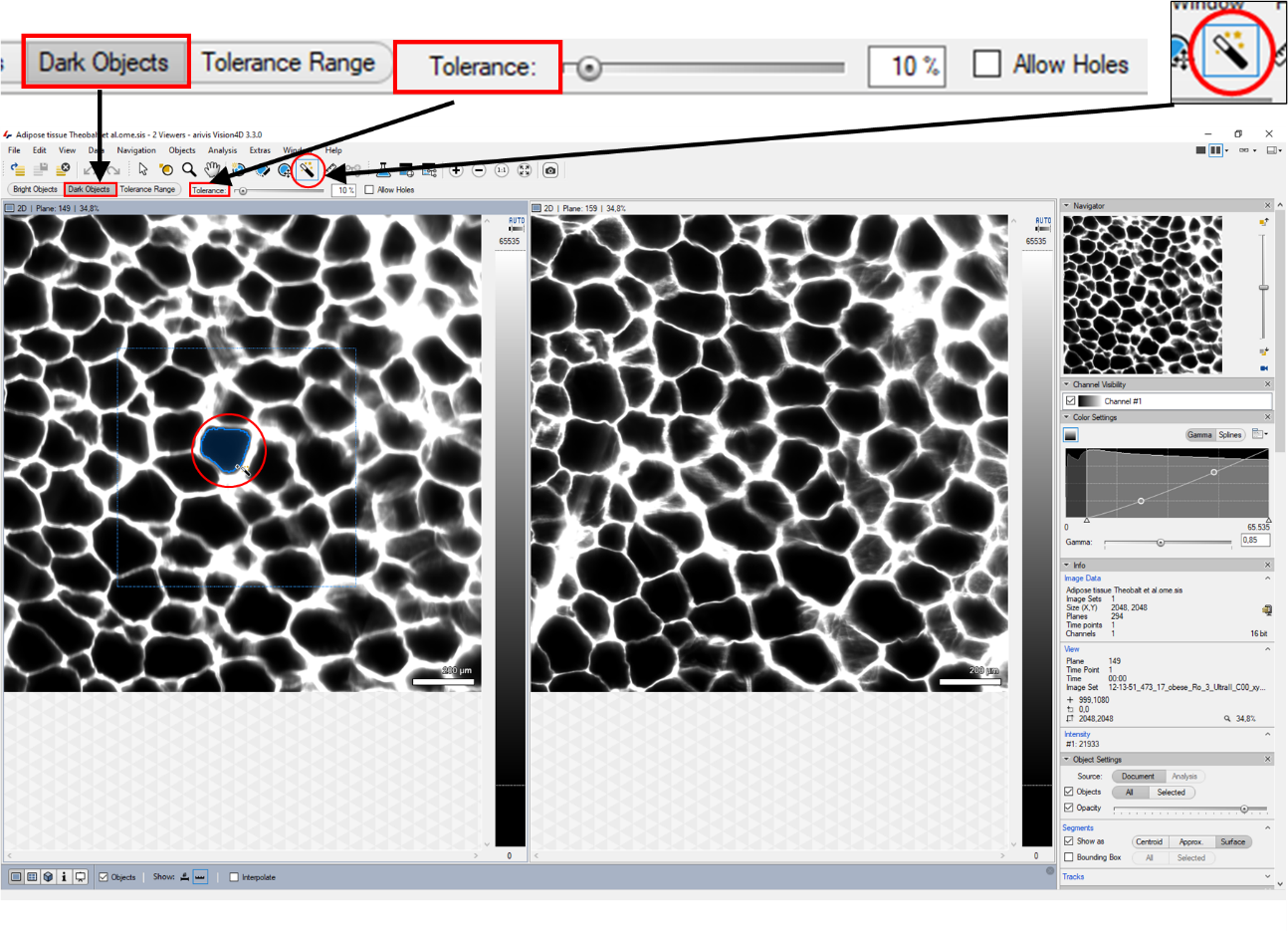
Screenshot 4.**

1. Select the “*Magic Wand*” tool (encircled in red, **Screenshot 4**) in the disector look up section image plane (N° 149, left screen side). Select the “*Magic Wand*” tool properties “*Dark Objects*” and a “*Tolerance*” of 10% (respectively of another appropriate tolerance level – use the same tolerance level for analysis of all images in all cases of a study). Move the mouse cursor over an adipocyte cross section profile (a square region of interest is shown around the mouse cursor and a preview of the section profile detected by the “*Magic Wand*” tool is displayed in a bluish color when hovering over it with the “*Magic Wand*” tool) and klick inside the black (unstained) center of the cell section profile (encircled in red).


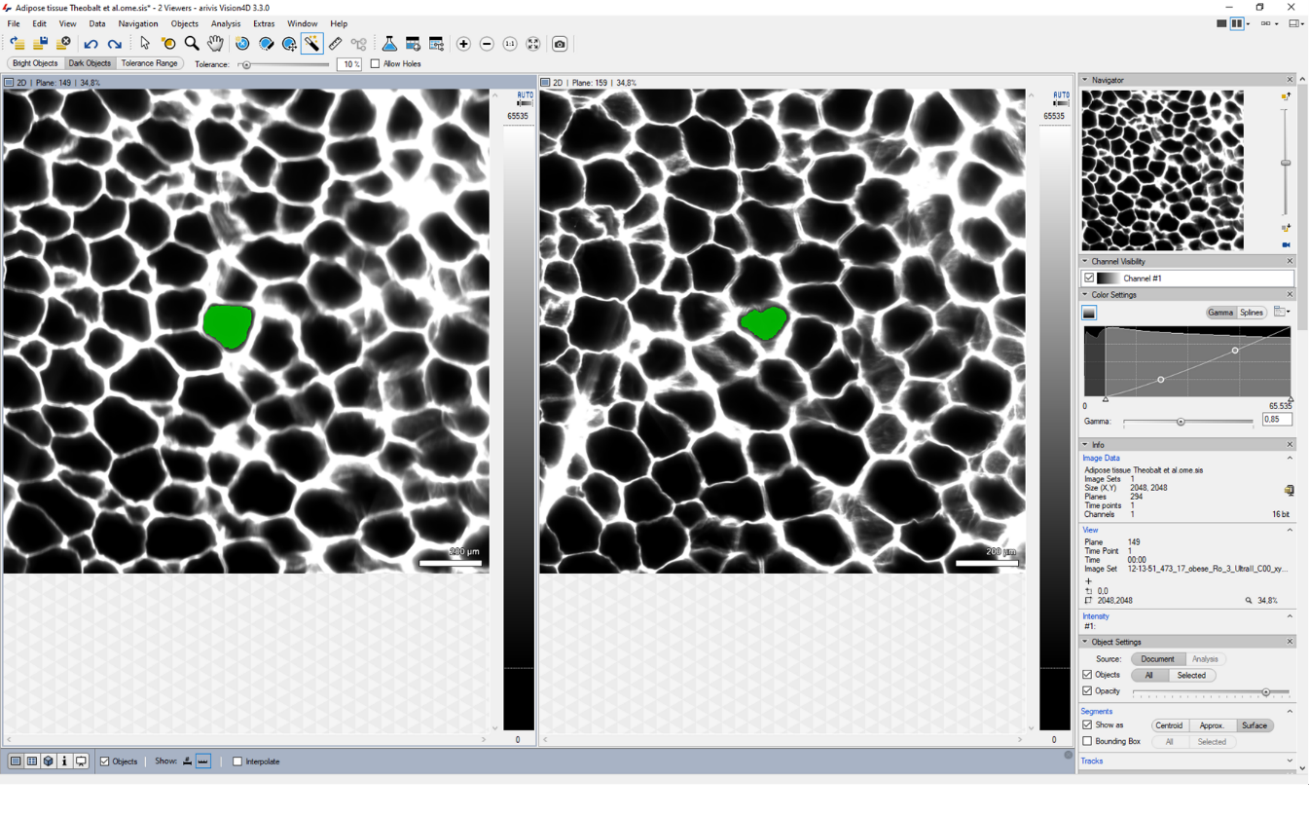
**Screenshot 5.**

1. The “*Magic Wand*” tool will automatically recognize the outer boundaries of the adipocyte cell membrane and label the adipocyte cell section profile areas in all image section levels of that single adipocyte with an individual color, *i.e.,* the profile of the adipocyte tagged in section plane N° 149 will automatically be tagged in the same color in section plane N° 159 (**Screenshot 5**) on right screen side (as well as in all other section planes).


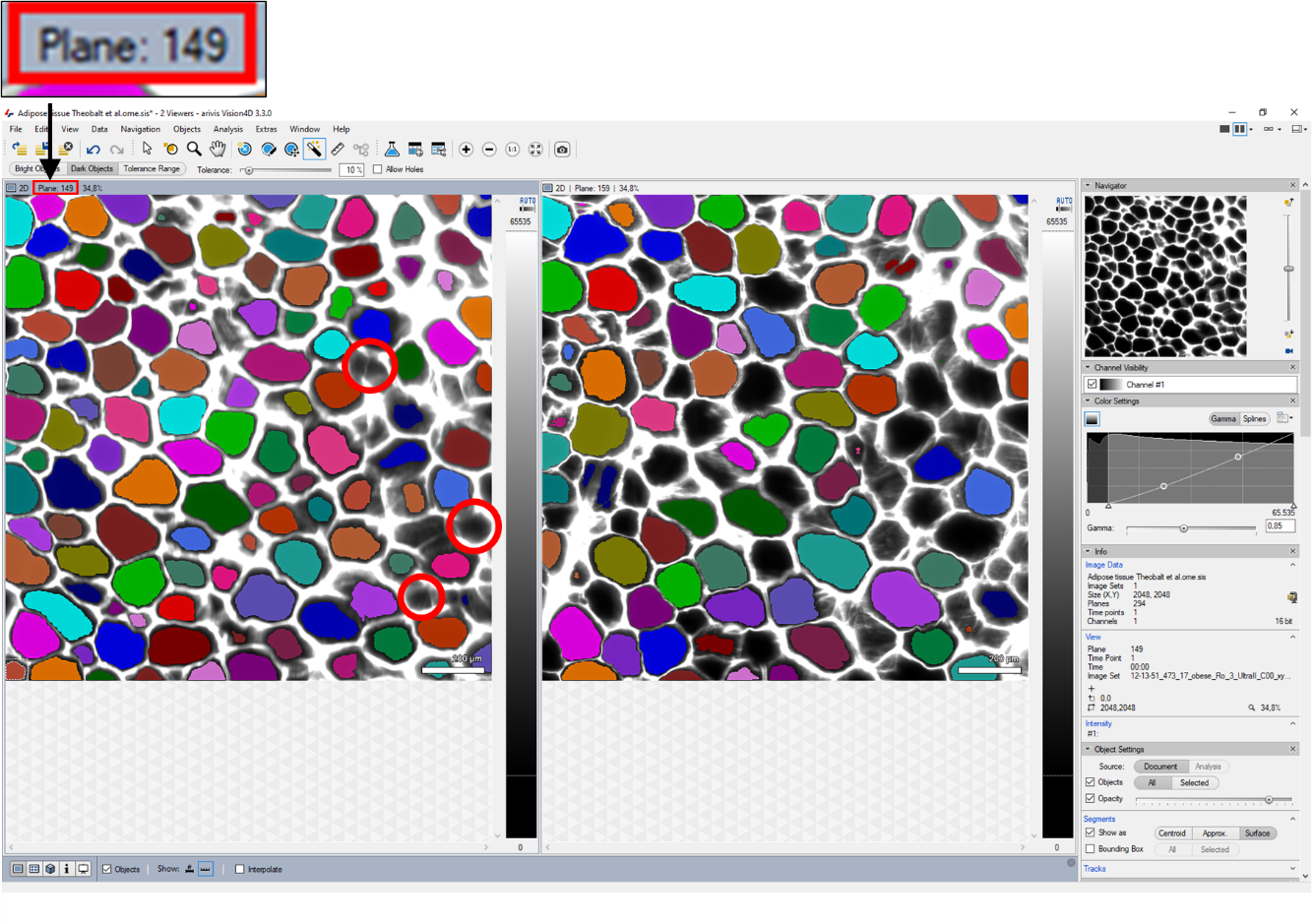
**Screenshot 6.**

1. Tag all adipocyte cross section profiles present in the look up section plane (section plane N° 149, **Screenshot 6**). Adipocytes also sectioned in the reference section plane (image plane N° 159) will automatically appear labeled in the corresponding color. Some adipocyte cross section profiles might apparently remain unlabeled (encircled in red) in the look up section plane (here: plane N° 149) although being tagged with the “*Magic Wand*” tool (**Screenshot 6**). By moving the slider of the “*Navigator*” window it can be controlled if the corresponding cell sections are correctly identified and labelled in upper/lower image section levels. **Screenshot 7** shows image section plane N° 139, where the cell section profiles of adipocytes which were apparently not labelled in section plane N° 149, are correctly labelled (encircled in red).

**
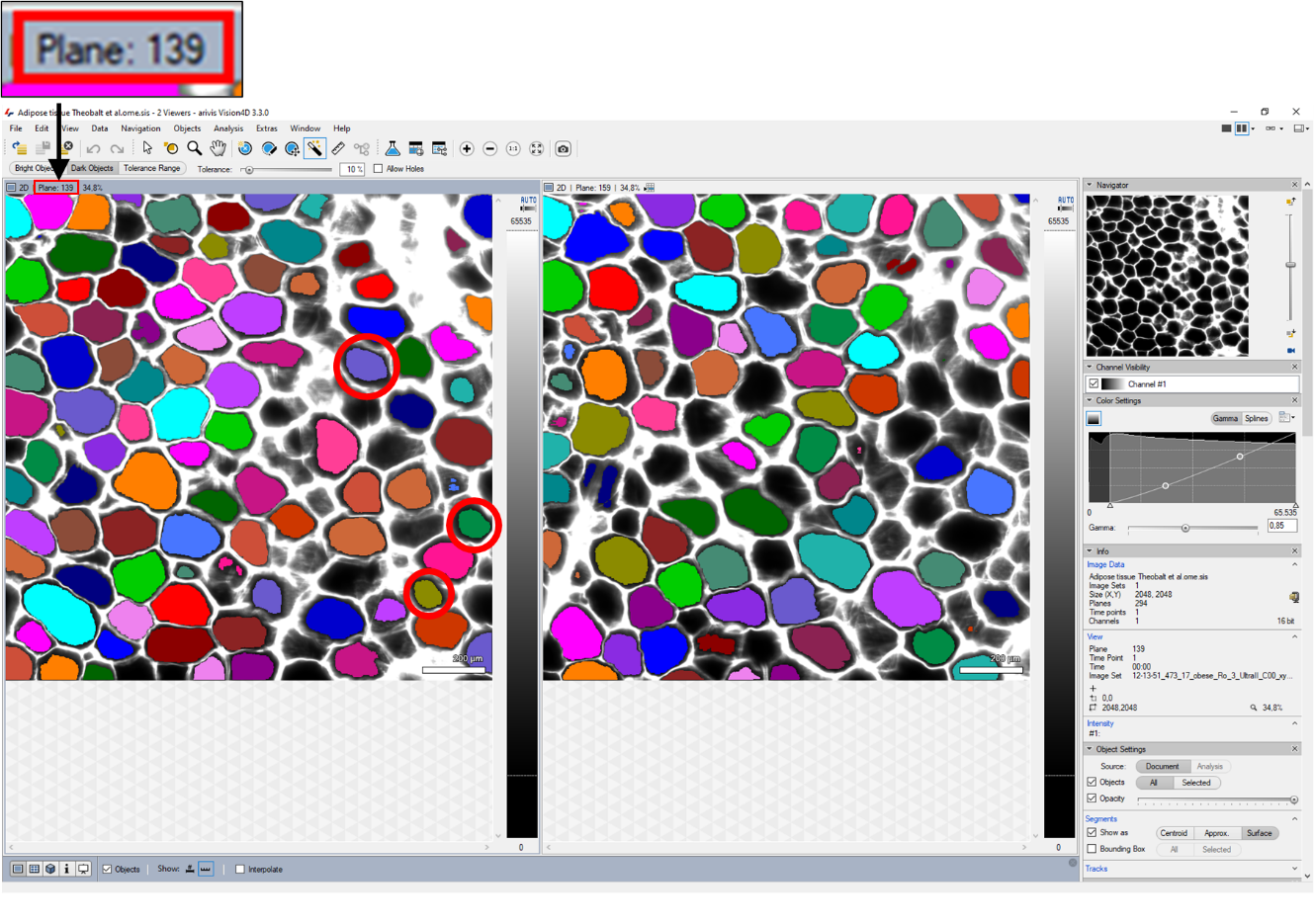
**

**Screenshot 7.**

**
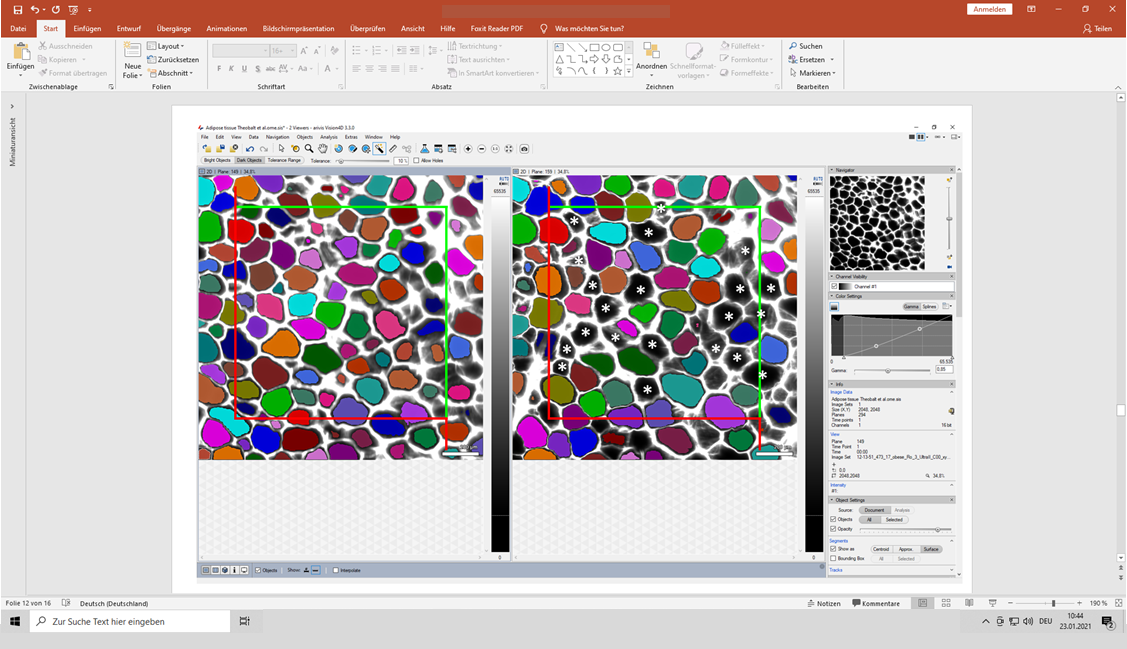
**

**Screenshot 8.**

1. When all adipocyte cross section profiles present in the look up section plane are tagged with the “*Magic Wand*” tool take a screenshot, copy it into another software-application (*e.g.,* Microsoft PowerPoint), and superimpose an unbiased counting frame (of known area) over a random location of the look up section plane (left screen side). Then also superimpose a second, equally sized unbiased counting frames over the corresponding location of the reference section (right screen side). [The arivis Vision4D imaging and analysis software does not provide a tool for superimposing unbiased counting frames]. Here, the area of the unbiased counting frame is 1165 x 1165 µm² (only relevant if numerical volume densities of adipocytes in the adipose tissue are determined with the disector method). Adipocytes present in the reference section plane but not in the look-up section plane are sampled with the unbiased counting frame (indicated by asterisks), using the (green) “inclusion lines” and (red) “exclusion lines” (**Screenshot 8**).

**
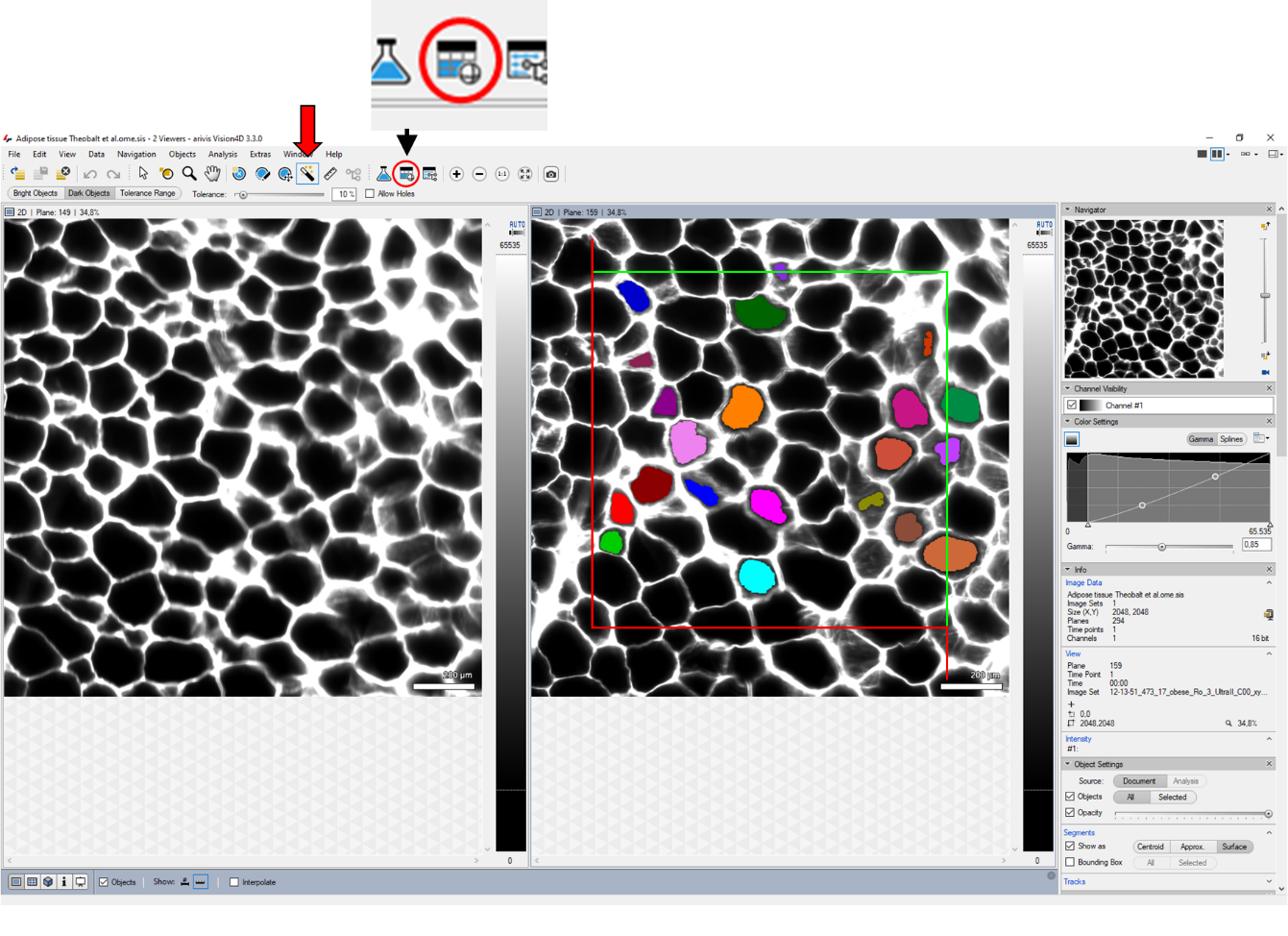
Screenshot 9.**

1. Move back to the arivis-window. Select the “*Show Objects Table*” button (encircled in red) and mark and delete all so-far tagged “*Magic Wand Objects*” (see III.3, **Screenshot 11**). Then again use the “*Magic Wand*” tool (arrow) to tag the section profiles of the adipocytes in the disector reference section plane sampled with the unbiased counting frame (indicated by asterisks in II.8, **Screenshot 8**) in the previous step. For better visualization, the unbiased counting frame is also superimposed here. In the present disector, 21 adipocytes are sampled.

_________________________________________________________________________________

**III. Direct 3D digital image analysis of individual adipocyte volumes**

*The “Magic Wand” tool is used to directly analyze volumes and other quantitative morphological parameters of the sampled adipocytes. To monitor the correct labelling/tagging of individual adipocytes (i.e., to verify that the analyzed cells are adipocytes, and that only individual adipocytes are analyzed), cells tagged with the “Magic Wand” tool can be inspected in the 3D view of the imaged tissue sample.*

1. With the reference section plane being active, select the 3D image display (red circle, red arrow in detail enlargement) to view the sampled adipocytes in 3D (**Screenshot 10, A**). Tick “*Objects*” and select “*Surface*” (red circle) for the display of 3D objects (**1**). Move the slider of the control row (**2**) down to see the “*4D Clipping”* panel (**Screenshot 10, B, 3**).


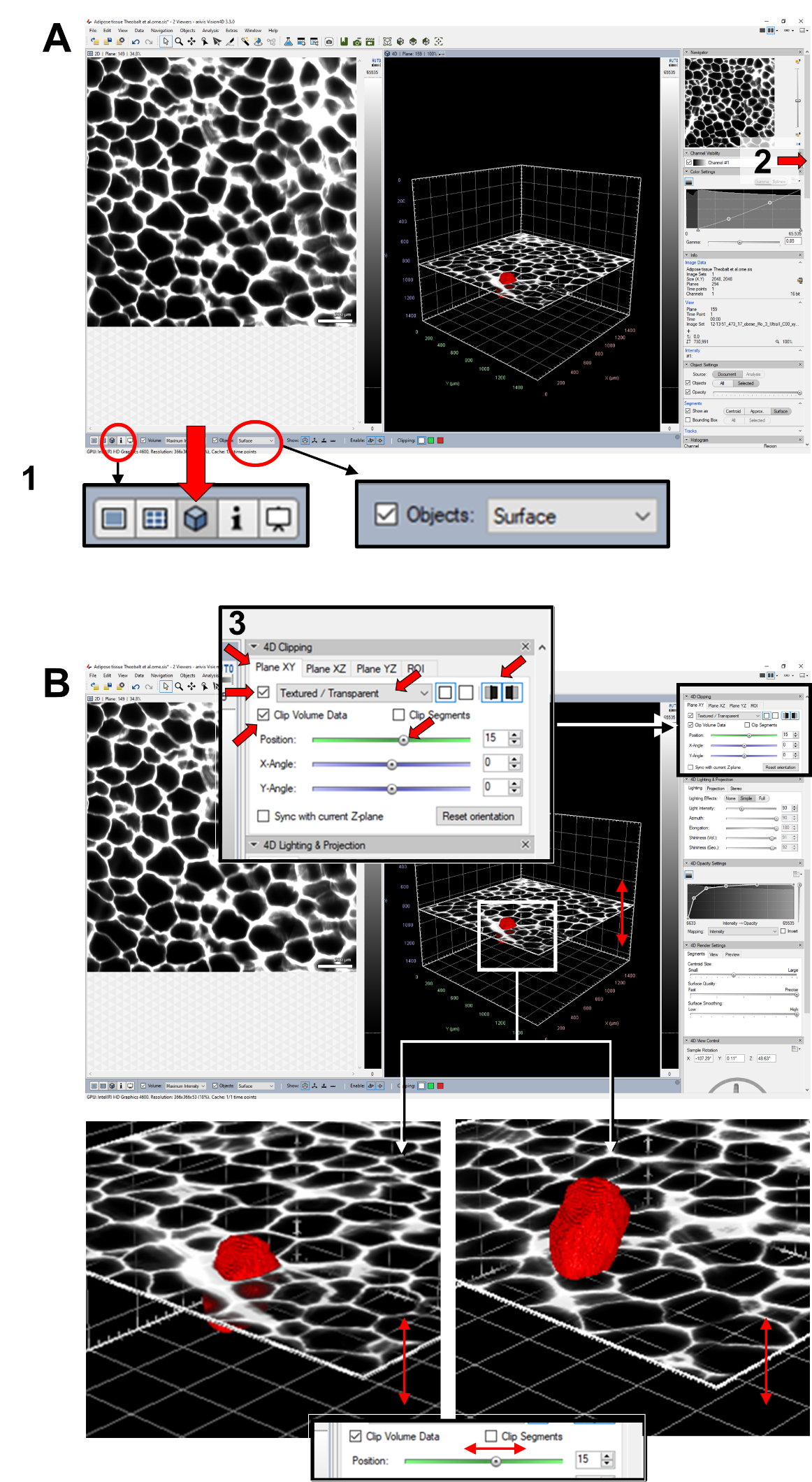


**Screenshot 10.**

1. To view individual adipocytes tagged with the “*Magic Wand*”-tool in 3D, select adipocytes to be displayed in the 3D view from the “*Objects Table*” (III.3, **Screenshot 11**). In **Screenshot 10 B**, the adipocyte (tagged in red color) sampled in the 2D view in **Screenshot 9** is displayed in the 3D-view. For better visualization, use the “*4D Clipping*” panel (**3**) to display a digital clipping plane through the tissue sample, using the indicated settings (red arrows). The position of the clipping plane can be varied, using the “*Position*”-slider. The 3D image of the adipocyte (*i.e.,* the “*Magic Wand Object*”) can be freely rotated and viewed from different perspectives. Use this tool to control the completeness of the 3D-labelling of the adipocyte in relation to its cell membrane boundaries (compare to Supporting Information **S1 video**). Control any sampled adipocytes in this way.


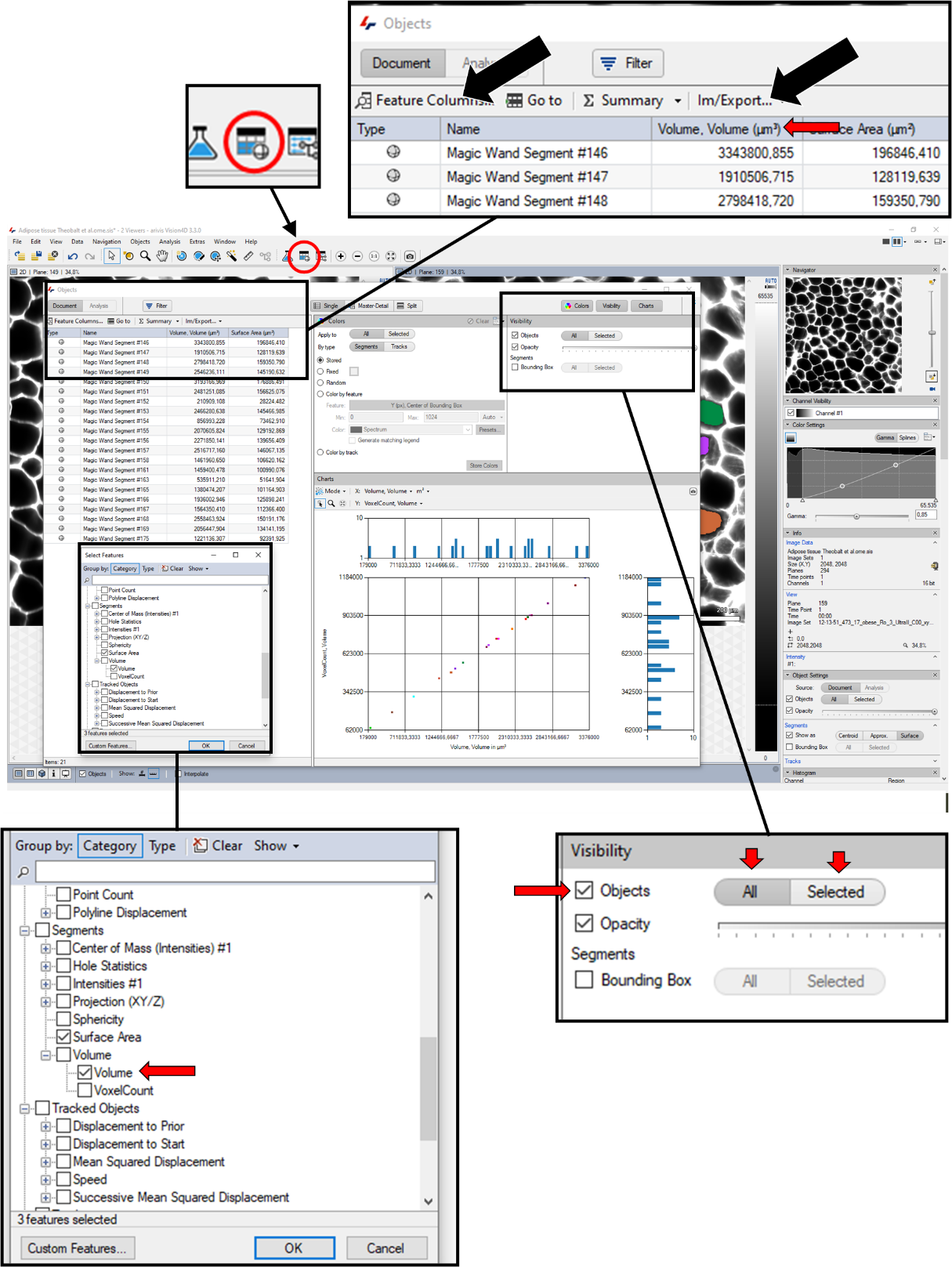


**Screenshot 11.**

1. For selection and analysis of morphometric parameters of unbiasedly sampled adipocytes tagged with the “*Magic Wand*” tool (*i.e.,* “*Magic Wand Objects*”) klick the “*Show Objects Table*” button (encircled in red, **Screenshot 11**). The “*Objects Table*” (inset) shows all “*Magic Wand Objects*” (titled as “*Magic Wand Segments*”) with their associated morphometric measurement results. The analyzed morphometric parameters can be selected from the “*Feature Columns*” menu (black arrow). In the present example, the parameters of “*Volume*” (red arrow) and “*Surface*” were selected (inset). Tagged adipocytes (*i.e., “Magic Wand Objects”*) can be selected (or deleted) individually in the “*Objects Table*”, as groups, or all. Selected “*Magic Wand Objects*” are correspondingly displayed in the 2D/3D viewer(s), as set in the “*Visibility*”-window (inset, red arrows). The analysis results can be exported from the “*Objects Table*” menu (*e.g.,* to Microsoft EXCEL), using the “*Im/Export*”-function (black arrow).
