# Supplementary material for: Unbiased analysis of obesity related, fat depot specific changes of adipocyte volumes and numbers using light sheet fluorescence microscopy
Source: PLoS One. 2021 Mar 16;16(3):e0248594. doi: 10.1371/journal.pone.0248594 (PMC7963095; doi:10.1371/journal.pone.0248594)
Supplement: S2 File — Downloadable from: doi:10.5061/dryad.8gtht76nt. (DOCX) [file pone.0248594.s008.docx]

**S2 file. Exemplary LSFM-dataset. Downloadable from: doi:10.5061/dryad.8gtht76nt.** For practical exercise, a z-stack of 181 2D-LSFM-image files from the present study can be downloaded from the URL provided in the paper on the publishers homepage. LSFM images were acquired from a 3DISCO-cleared, eosin-stained, subcutaneous adipose tissue sample of an obese Göttingen minipig, exactly as described in the “Materials and Methods” section (Ex/Em: 520/40nm/585/40nm; z-step size = 5 µm).

**Literature**

1. Howard CV, Reed MG. Unbiased Stereology. 2 ed. Coleraine, UK: QTP Publications; 2005. 278 p.

2. Gundersen HJ, Jensen EB. The efficiency of systematic sampling in stereology and its prediction. J Microsc. 1987;147(Pt 3):229-63.

3. Albl B, Haesner S, Braun-Reichhart C, Streckel E, Renner S, Seeliger F, et al. Tissue sampling guides for porcine biomedical models. Toxicol Pathol. 2016;44(3):414-20.

4. Blutke A, Wanke R. Sampling strategies and processing of biobank tissue samples from porcine biomedical models. J Vis Exp. 2018(133).

5. Howard CV, Reed MG. Unbiased Stereology. 2 ed. Coleraine, UK: QTP Publications; 2005.

6. Delesse MA. Procédé mécanique pour déterminer la composition des roches. C R Acad Sci Paris 1847;25:544-5.

7. Weibel ER. Stereological methods. I. Practical methods for biologicalmorphometry. London: Academic Press; 1979.

8. Weibel ER. Stereological Methods II. Theoretical foundations. London: Academic Press; 1980.

9. Dorph-Petersen KA, Nyengaard JR, Gundersen HJ. Tissue shrinkage and unbiased stereological estimation of particle number and size. J Microsc. 2001;204(Pt 3):232-46.

10. Gundersen HJG, Mirabile R, Brown D, Boyce RW. Stereological principles and sampling procedures for toxicologic pathologists. In: Haschek WMea, editor. Haschek and Rousseaux´s Handbook of Toxicologic Pathology London: Academic Press. INC.; 2013. p. 215-86.

11. Tschanz S, Schneider JP, Knudsen L. Design-based stereology: Planning, volumetry and sampling are crucial steps for a successful study. Ann Anat. 2014;196(1):3-11.

12. Blutke A, Schneider MR, Wolf E, Wanke R. Growth hormone (GH)-transgenic insulin-like growth factor 1 (IGF1)-deficient mice allow dissociation of excess GH and IGF1 effects on glomerular and tubular growth. Physiol Rep. 2016;4(5):e12709.

13. Matenaers C, Popper B, Rieger A, Wanke R, Blutke A. Practicable methods for histological section thickness measurement in quantitative stereological analyses. PLoS One. 2018;13(2):e0192879.

14. Sterio DC. The unbiased estimation of number and sizes of arbitrary particles using the disector. J Microsc. 1984;134(Pt 2):127-36.

15. Mattfeld T. Stereologische Methoden in der Pathologie. Doerr W, Leonhardt H, editors. Stuttgart-New York: Georg Thieme Verlag; 1990.
